# Identification of cell surface markers and establishment of monolayer differentiation to retinal pigment epithelial cells

**DOI:** 10.1038/s41467-020-15326-5

**Published:** 2020-03-30

**Authors:** Alvaro Plaza Reyes, Sandra Petrus-Reurer, Sara Padrell Sánchez, Pankaj Kumar, Iyadh Douagi, Hammurabi Bartuma, Monica Aronsson, Sofie Westman, Emma Lardner, Helder André, Anna Falk, Emeline F. Nandrot, Anders Kvanta, Fredrik Lanner

**Affiliations:** 1 Department of Clinical Sciences, Intervention and Technology, Karolinska Institutet, Stockholm, Sweden; 2 Ming Wai Lau Center for Reparative Medicine, Stockholm node, Karolinska Institutet, Stockholm, Sweden; 3 Division of Obstetrics and Gynecology, Karolinska Universitetssjukhuset, Stockholm, Sweden; 4 Department of Clinical Neuroscience, Division of Eye and Vision, St. Erik Eye Hospital, Karolinska Institutet, Stockholm, Sweden; 5 Department of Medicine, Center for Hematology and Regenerative Medicine, Karolinska Institutet, Stockholm, Sweden; 6 Department of Neuroscience, Karolinska Institutet, Stockholm, Sweden; 7 INSERM, CNRS, Institut de la Vision, Sorbonne Université, 75012 Paris, France

**Keywords:** Embryonic stem cells, Stem-cell research

## Abstract

In vitro differentiation of human pluripotent stem cells into functional retinal pigment epithelial (RPE) cells provides a potentially unlimited source for cell based reparative therapy of age-related macular degeneration. Although the inherent pigmentation of the RPE cells have been useful to grossly evaluate differentiation efficiency and allowed manual isolation of pigmented structures, accurate quantification and automated isolation has been challenging. To address this issue, here we perform a comprehensive antibody screening and identify cell surface markers for RPE cells. We show that these markers can be used to isolate RPE cells during in vitro differentiation and to track, quantify and improve differentiation efficiency. Finally, these surface markers aided to develop a robust, direct and scalable monolayer differentiation protocol on human recombinant laminin-111 and −521 without the need for manual isolation.

## Abstract (web summary)

Whilst pigmentation has been used to identify retinal pigment epithelial (RPE) cells, surface markers for these cells remain unclear. Here, the authors define surface markers for the RPE including CD140b, which help produce hPSC-derived RPE cells at a large scale following a robust, direct and scalable monolayer differentiation protocol.

## Introduction

Age-related macular degeneration (AMD) is the major cause of severe vision loss in people over 60 years of age, with 500,000 new cases each year in the Western countries^1^. Up to date, AMD is estimated to affect 170 million worldwide, a number predicted to increase to 196 million in the coming 5 years and up to 288 million in 2040^2^, implying substantial social and financial consequences. AMD comes in two forms: neovascular or “wet” AMD, characterized by the abnormal growth of choroidal vessels through the Bruch’s membrane causing subretinal edema and hemorrhage; and “dry” AMD, which in advanced stages is characterized by well demarcated areas of RPE loss and outer retinal degeneration, also known as geographic atrophy^3,4^. The “dry” form accommodates 80–90% of the AMD patients, and although neovascular AMD is currently treated with anti-vascular endothelial growth factor injections, there is no treatment available for “dry” AMD patients. Therefore, subretinal transplantation of RPE cells derived from human pluripotent stem cells (hPSCs) emerges as a potential replacement therapy in geographic atrophy. Although there are several protocols describing the derivation of RPE from a hPSC source, most of them still rely on the manual selection of pigmented patches of cells to reach higher purity^5–11^. Such manual selection makes large-scale production of hPSC-RPE cells cumbersome and carries a potential risk of tumorigenicity, if residual undifferentiated cells remain undetected in the final product. From this perspective, it would be useful to have cell surface markers, which would allow both prospective isolation of hPSC-RPE in an automated manner and also quantitative analysis of RPE purity and absence of unwanted cell types, such as undifferentiated cells and alternative lineages that could emerge during the differentiation process. In contrast to RPE, such cell surface markers have been identified for undifferentiated and many differentiated cell types, including cardiac, pancreatic, and neural lineages, proving to be highly useful in eliminating undifferentiated cells, tracking, optimizing, and aiding differentiation, as well as in predicting transplantation outcome^12–16^.

In the present study, we identify CD140b, CD56, GD2, and CD184 as central cell surface markers to evaluate hPSC-RPE differentiation efficiency, as well as a potential tool for the enrichment of hPSC-RPE during and after differentiation. Using these markers together with single-cell RNA-sequencing to evaluate the differentiation process, we have established an efficient xeno-free and defined monolayer differentiation methodology, where culture on supportive human recombinant laminin (hrLN) eliminates the need for manual selection, allowing large-scale production of pure hPSC-RPE.

## Results

### Identification of CD140b^+^GD2^−^ and CD184^−^ as hPSC-RPE markers

**Fig. 1:**
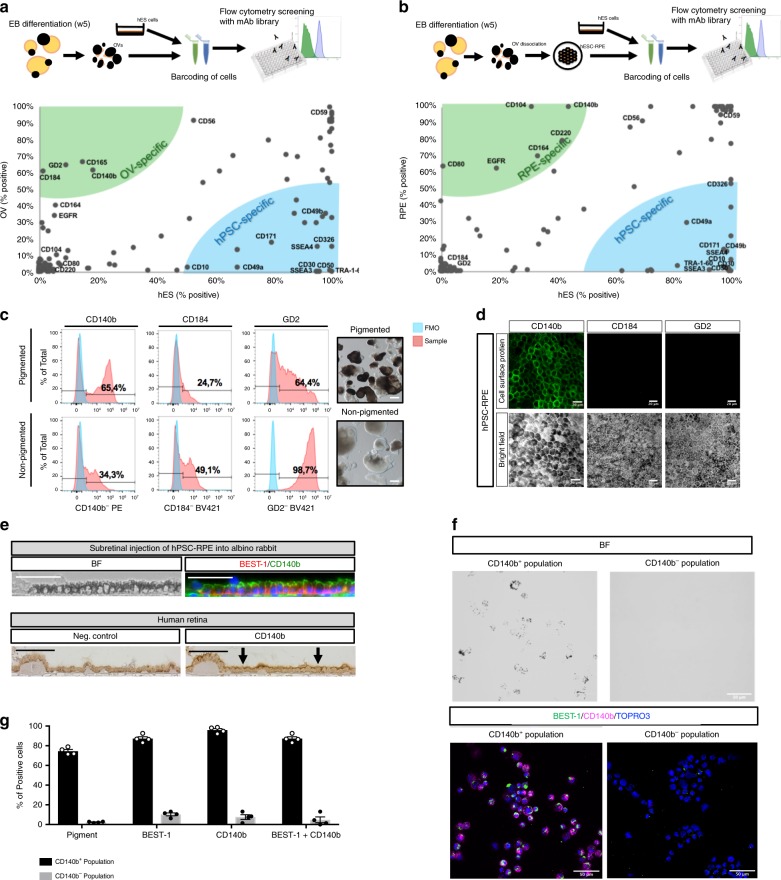
hPSC-RPE cell surface marker screening and validation. **a**, **b** Schematics of the antibody library screen and dot-plot graphs displaying the most relevant markers identified with the antibody library and their relative degree of expression between the hESC and optic vesicle (OV) cell populations (**a**) and between the hESC and day 60 hPSC-RPE populations (**b**). Each dot represents a different cell surface protein, and their position along the *x* and *y* axes is determined by the percent positive value in hESC and optic vesicle-cell/hPSC-RPE samples. Based on their position in the chart, a subset of cell surface proteins have been categorized as hPSC specific (bottom-right region) or optic vesicle specific (top-left region). **c** Flow cytometry histograms representing percentage of positive cells for CD140b, GD2, and CD184 in the pigmented and non-pigmented fractions of the embryoid bodies after 30 days of differentiation. Representative bright field pictures depicting the pigmented and non-pigmented fractions of the embryoid bodies that were analyzed by flow cytometry. Negative gates were set based on fluorescence minus one (FMO) control samples. Results are based on pooled samples from three independent differentiations. **d** Immunofluorescence stainings displaying the expression pattern of CD140b, CD184, and GD2 cell surface markers in day 60 hPSC-RPE cells. **e** Upper: Bright field and immunofluorescent pictures displaying the expression pattern of CD140b and human-specific BEST-1 (does not label rabbit BEST-1) in albino rabbit subretinally injected with hPSC-RPE cells. Pigmentation is of human origin as albino rabbits lack pigmentation. Lower: Bright field immunohistochemistry pictures showing the expression of CD140b in a human subretinal tissue section. **f** Bright field and immunofluorescent images showing pigmentation, as well as BEST-1 and CD140b co-expression patterns in the CD140b^+^ and CD140b^−^ populations sorted at day 30 of differentiation. **g** Bar graphs representing the quantification of cells that are pigmented, BEST-1^+^, CD140b^+^, BEST-1 and CD140b^+^ in the CD140b^+^ and CD140b^−^ sorted populations. Bars represent means ± SEM from four different images. Scale bars: **c** = 200 μm; **d** = 20 μm; **e**, **f** = 50 μm. Source data are provided as a Source Data file.

With the aim of finding new surface markers for hPSC-RPE, we used our published protocol to differentiate hPSC into RPE cells using 3D embryoid body (EB) differentiation^10,17^. After 3 weeks of culture, optical vesicles emerge from the EBs containing the pigmented RPE cells mixed with other cell types. Optic vesicles were manually isolated at 5 weeks and expression of cell surface markers was compared with undifferentiated human embryonic stem cells (hESC) using an antibody library recognizing 242 CD antigens with flow cytometry (Fig. 1a and Supplementary Fig. 1a, b). The screening identified subsets of cell surface markers expressed in both cell types and specific for either the optic vesicles or the hESC. Even though CD59 has been suggested to be a useful RPE marker^18^, our screening and immunostaining data show that it is expressed in both RPE and undifferentiated embryonic stem cells, thus making it less suitable (Fig. 1a, b and Supplementary Fig. 1c). In agreement with optic vesicles consisting of several cell types, we did not detect homogeneous labeling of all cells in the optic vesicle sample. Instead, the optic vesicle-specific markers labeled a fraction of the cells, suggesting that they potentially tag different cell types within the dissected optic vesicles. To identify markers that could possibly be RPE specific and those that may label alternative lineages, we performed a second screening, this time using more mature end-stage differentiation cultures coming from dissociated optic vesicles that were cultured for an additional 30 days on hrLN-521, which we previously showed to be highly pure and functional^10^ (Fig. 1b). Compiling the results from both screenings, we found CD140b (recognizing platelet-derived growth factor receptor-β; PDGFRB) as the only marker that appeared already at the optic vesicle stage and remained highly expressed in the later differentiation stage, identifying CD140b as a potential marker of both early and more mature hPSC-RPE cells. The second screen identified additional markers that specifically labeled the more mature and pure hPSC-RPE cells, such as CD104 (recognizing integrin β4 chain, which is required for hemidesmosome formation in epithelial cells)^19^, but neither of these markers were detected in a significant fraction of the RPE in the early optical vesicle stage, suggesting that these would be less useful to track emergence of RPE cells. Besides CD140b, we found GD2 (recognizing Disialoganglioside GD2) and CD184 (recognizing C–X–C motif chemokine receptor 4) markers labeling a large fraction of cells at the optic vesicle stage while absent at the later stages, suggesting that they may label alternative lineages in the early cultures (all screening data summarized in Supplementary Data 1). In agreement with this finding, CD140b proved to be more restricted to the dissected pigmented optic vesicles over the remaining non-pigmented EB structures, whereas GD2 and CD184 were detected at higher levels in the non-pigmented structures (Fig. 1c). Additionally, CD140b^+^ cells were present in mature hPSC-RPE cultures, while GD2- and CD184-expressing cells were completely absent, as immunofluorescence staining confirmed on hPSC-RPE cells in culture (Fig. 1d). To ensure specificity of CD140b as a cell surface marker of hPSC-RPE, day 30 CD140b^+^ and CD140b^−^ cell populations present in hPSC-RPE cultures were isolated through fluorescence-activated cell sorting (FACS sorting) (Supplementary Fig. 1d). Only the CD140b^+^ cell fraction was pigmented, co-stained positively for BEST-1 (Bestrophin 1), and showed higher expression levels of RPE-specific markers such as *TYR*, *BEST-1*, and *RPE65*, with lower expression levels of pluripotency and neuronal markers, including *POU5F1*, *MAP2*, and *TUBB3* (Fig. 1f–g and Supplementary Fig. 1e). Following 30 days in culture, only the CD140b^+^, but not the negative cell fraction, expanded into hPSC-RPE cells displaying a cobblestone and homogeneous morphology (Supplementary Fig. 1f). Finally, we assessed the presence of CD140b in the in vivo retina. Histology of transplanted hPSC-RPE into albino rabbit subretina (lacking endogenous pigmentation of the RPE) showed apical expression of CD140b and basal expression of BEST-1 on pigmented hPSC-RPE cells (using human-specific BEST-1 antibody). The apical expression of CD140b was confirmed also by immunohistochemistry in adult human RPE (Fig. 1e), in agreement with the expression pattern in the mouse^20^.

### Monolayer differentiation on hrLN

**Fig. 2:**
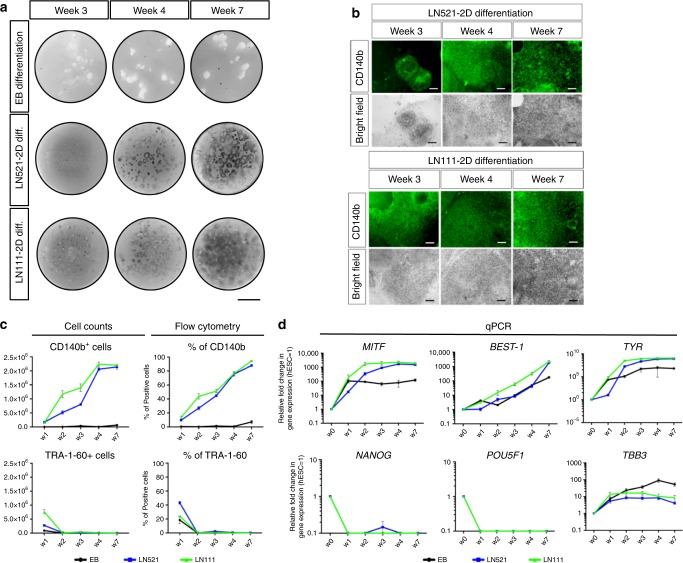
hPSC-RPE induction in 2D using hrLN-111 and hrLN-521. **a** Camera pictures showing the progression of the differentiation (note pigmentation, weeks 3–7) in suspension cultures (embryonic bodies, EB) and 2D in both hrLN-111 and hrLN-521. **b** Bright field and immunofluorescence pictures showing the increase of CD140b expression and pigmentation level during the time course of 2D hPSC-RPE differentiation on hrLN-521 and hrLN-111. **c** Charts comparing the yield and percentage of positive cells, measured by flow cytometry, for CD140b and TRA-1-60 during the time course of hPSC-RPE differentiation among the three different protocols tested (embryoid bodies, hrLN-521 and hrLN-111). **d** Gene expression analysis of pluripotency and RPE-specific genes throughout the time course showed in **a**. Values are normalized to *GAPDH* and displayed as relative to undifferentiated hESC. Bars represent means ± SEM from three independent experiments. Scale bars: **a** = 5 mm; **b** = 100 μm. Source data are provided as a Source Data file.

We recently developed a xeno-free and defined hPSC-RPE differentiation methodology using suspension EB differentiation to induce the RPE cell fate^10^. However, due to the significant variability between experiments and starting cell lines, we decided to evaluate whether translating this protocol into a 2D monolayer culture would facilitate better reproducibility. For this reason, we tested two human substrates present in the endogenous Bruch’s membrane: hrLN-111 and hrLN-521^21,22^. hESC were plated at a cell density of 2.4 × 10^4^ cells/cm^2^ on both substrates and evaluated 30 days after plating. Prominent pigmentation was observed on both substrates (Supplementary Fig. 2a). In agreement with previous studies suggesting that Activin A is a potent retinal fate inducer^23–26^, we observed significant increase of pigmentation together with corresponding transcriptional maturation towards RPE fate with addition of Activin A (Supplementary Fig. 2a–c). Flow cytometry analysis using our identified extracelullar markers supported this result with increased CD140b^+^ fraction from ~40 to 90%, with Activin A on both substrates (Supplementary Fig. 2d). Next, we compared monolayer differentiation with our previously established suspension differentiation as EBs^10^. While suspension EB cultures did generate some pigmented structures as described before, the monolayers were dominated by pigmented cells following 7 weeks of differentiation (Fig. 2a). This increase was mirrored in CD140b protein expression through time (Fig. 2b). Evaluating purity based on pigmented cells is difficult, particularly in EB cultures, but quantification of CD140b^+^ cells indicated that EB cultures contain ~10% of prospective RPE cells, while the monolayer laminin cultures reached levels of 90% (Fig. 2c). Additionally, transcriptional dynamics showed approximately ten times increase in RPE-associated transcripts, such as *MITF* (microphthalmia-associated transcription factor), *BEST-1*, and *TYR*, while reducing expression of neuronal transcript *TBB3* on both laminins compared to suspension EB differentiation (Fig. 2d).

### Replating gives high yield of pure and functional hPSC-RPE

**Fig. 3:**
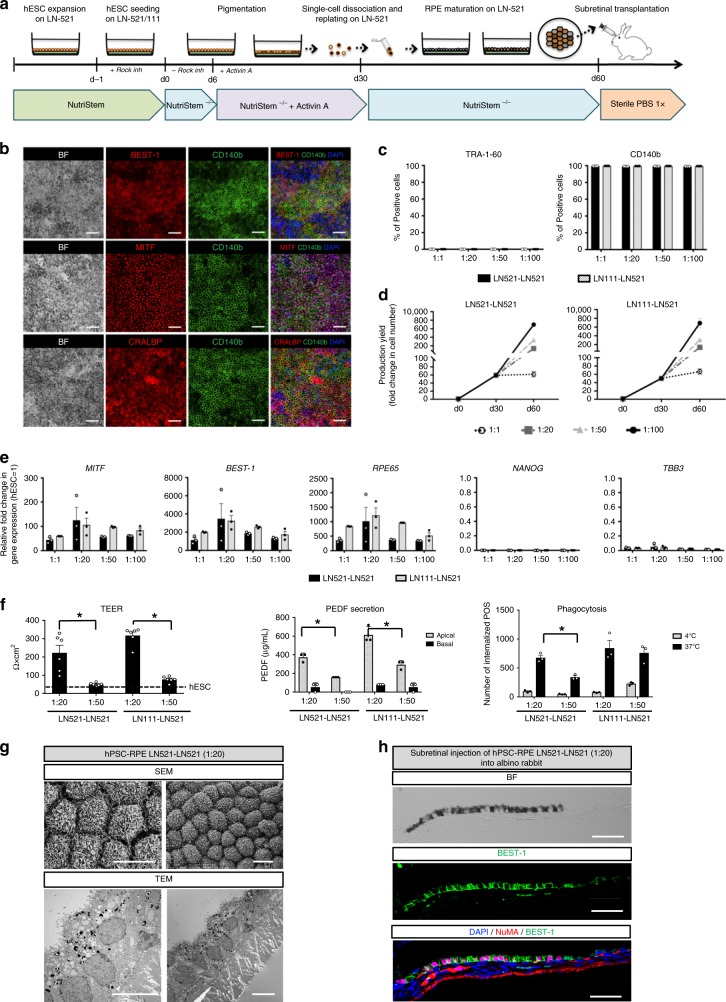
Expansion and enrichment of monolayer differentiated hPSC-RPE on hrLN-521. **a** hPSC-RPE monoloayer differentiation protocol scheme (top image was adapted from ref. ^10^, Copyright (2016), with permission from Elsevier). hESC cultures are seeded on hrLN-521- or hrLN-111-coated plates in NutriStem hPSC XF medium containing Rho-kinase inhibitor (Y-27632) in 5% O_2_. After 24 h, the medium is replaced with differentiation medium (NutriStem hPSC XF without bFGF and TGFβ) and cells are placed in 21% O_2_. From day 6 after plating, 100 ng/mL of Activin A (R&D Systems) is added to the media until day 30. Cells are then enzymatically dissociated, replated on hrLN-521, and cultured until homogeneous pigmentation is reached (day 60). At that point, single-cell suspension is injected subretinally in the rabbit eye. **b** Bright field and immunofluorescent images showing the co-expression of RPE-specific markers (BEST-1, MITF, CRALBP) with CD140b in hPSC-RPE day 60. **c** Flow cytometry for TRA-1-60 and CD140b following replating at cell densities: 1.4 × 10^6^ cells/cm^2^ (1:1), 7 × 10^4^ cells/cm^2^ (1:20), 2.8 × 10^4^ cells/cm^2^ (1:50), and 1.4 × 10^4^ cells/cm^2^ (1:100). **d** Relative cell yield during the differentiation protocol at various replating densities. **e** Gene expression analysis of pluripotency, RPE- and neural-specific genes normalized to *GAPDH* and relative to hESC. **f** Functional assays demonstrating epithelial integrity by transepithelial resistance (TEER), PEDF secretion by ELISA, and internalization of photoreceptor outer segments (POS). The TEER value for hESC is shown for comparison (dashed line). **g** SEM and TEM images of hESC-RPE cultured on hrLN-521 at two different magnifications showing surface microvilli and polarized intracellular structures. **h** Pigmented monolayer formation in albino rabbits demonstrated by bright field imaging and immunofluorescence of anti-human BEST-1 and NuMA upon subretinal injection of hPSC-RPE cultured in hrLN-521 (replated at a cell density of 7 × 10^4^ cells/cm^2^). Bars represent means ± SEM from three independent experiments. (*) Asterisks represent significance with a *P* value < 0.0001 (3F, TEER, and PEDF secretion); =0.0042 (3F, Phagocytosis). Scale bars: **b** = 20 μm; **g** = 10 μm; **h** = 50 μm. Source data are provided as a Source Data file.

As the laminin monolayer cultures were permissive for hPSC-RPE differentiation with >80% of the cells being CD140b^+^ at 4 and 7 weeks, we explored whether a purer final hPSC-RPE product could be achieved by introducing a replating strategy. This seemed feasible as we found in the previous protocol that replating of dissociated optic vesicles, which contain multiple lineages, onto hrLN-521 would selectively support RPE cells to grow and produce more than 95% pure cultures^10^. Therefore, directly after 30 days of hPSC-RPE monolayer differentiation on hrLN-111 or hrLN-521, we dissociated the cultures to single-cell suspension and plated them on hrLN-521 (Fig. 3a) at four different dilutions 1:1 (1.4 × 10^6^ cells/cm^2^), 1:20 (7 × 10^4^ cells/cm^2^), 1:50 (2.8 × 10^4^ cells/cm^2^), and 1:100 (1.4 × 10^4^ cells/cm^2^). Following an additional 30 days, we observed robustly pigmented cultures of hexagonal epithelial cells that also co-expressed CD140b with BEST-1, MITF, and CRALBP (Fig. 3b and Supplementary Fig. 3a). Flow cytometry analysis showed homogeneous cultures of hPSC-RPE, now with >99% of the cells being CD140b^+^ and TRA-1-60^−^ (Fig. 3c). The final yield of CD140b^+^ hPSC-RPE cells followed the increasing dilutions ranging from 50- to 8000-fold yield relative to the number of starting pluripotent stem cells (Fig. 3d). Transcriptional analysis also showed robust induction of RPE-related markers, such as *MITF*, *BEST-1*, and RPE65, and down-regulation of pluripotency and neuronal transcripts *NANOG* and *TBB3* with similar patterns in all dilutions independently of the initial laminin coating (Fig. 3e).

Functional analysis of epithelial function measured by transepithelial resistance (TEER), polarized secretion of pigment epithelium-derived factor (PEDF) measured by enzyme-linked immunosorbent assay (ELISA), and phagocytosis of photoreceptor outer segments (POSs) show significantly higher functionality in 1:20 dilution, suggesting that this would be the optimal replating density (Fig. 3f and Supplementary Fig. 3b), with a final yield of 1300-fold increase compared to the starting number of hPSCs. Scanning and transmission electron microscopy of hPSC-RPE replated in this optimal density revealed extensive apical microvilli and a polarized intracellular localization of melanosomes towards the apical side, which further confirms the differentiated state of hPSC-RPE cells (Fig. 3g and Supplementary Fig. 3c). Lastly, bright field and immunofluorescence of transplanted albino rabbits showed subretinal monolayer formation of pigmented hPSC-RPE with basal expression of human BEST-1 (Fig. 3h).

### Single-cell RNA-sequencing shows high purity of hPSC-RPE

Although replating the monolayer cultures did generate seemingly pure hPSC-RPE cells, we still wondered whether enrichment using our identified cell surface markers could further improve the purity of the final product. We also wanted to ensure that we did not have any contaminating pluripotent cells or other alternative cell type in our final culture with or without the use of cell enrichment.

**Fig. 4:**
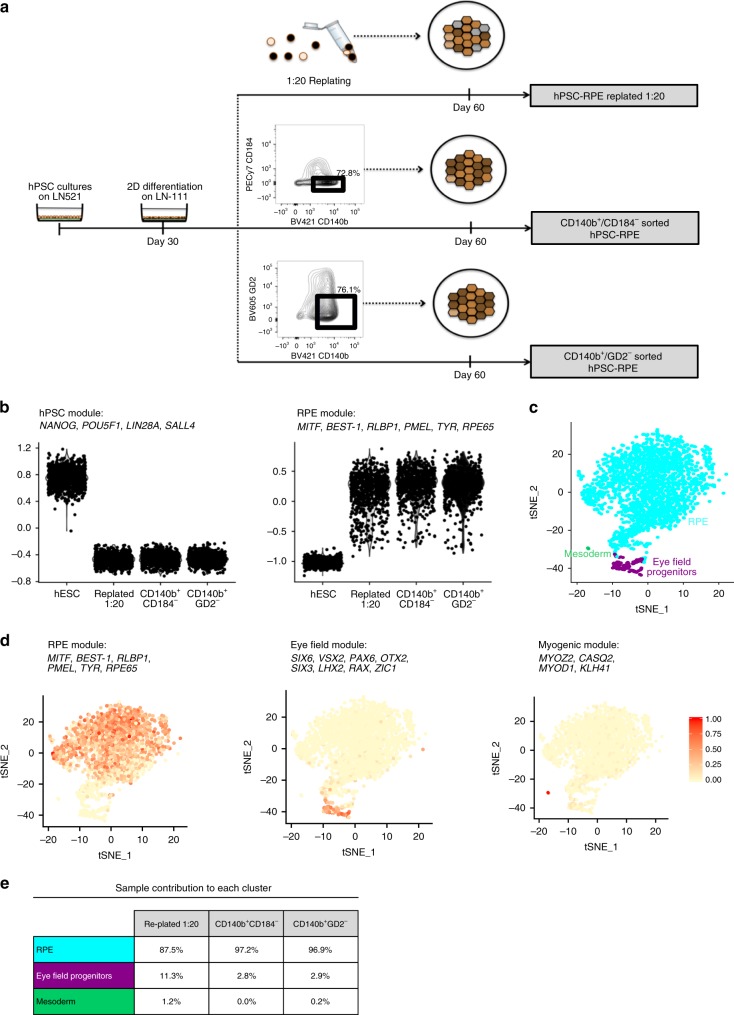
Single-cell RNA-sequencing analysis following monolayer differentiation with and without cell surface marker enrichment. **a** Schematics representing the differentiation routines for the FACS-sorted and replated hPSC-RPE cells that will be analyzed by 10x Genomics scRNA-seq. Included in the schematics, representative density plots illustrating the FACS sorting strategy followed to separate CD140b^+^/CD184^−^ and CD140b^+^/GD2^−^ cells at day 30 of hPSC-RPE differentiation. These sorted populations were subsequently seeded and cultured on hrLN-521 until day 60 of differentiation, when they were analyzed by 10x Genomics scRNA-seq. **b** Violin plots representing module expression scores for distinctive genes of hPSC (*POU5F1*, *NANOG*, *LIN28A*, and *SALL4*) and RPE cells (*MITF*, *BEST-1*, *RLBP1*, *PMEL*, *TYR*, *RPE65*). **c** tSNE representation of all cells showing cell lineage assignment of cells, using the 797 most variable genes across all cells. Cells are colored according to cluster and inferred cell type. **d** Feature plots displaying expression module scores over the tSNE plot for distinctive genes of RPE cells (*MITF*, *BEST-1*, *RLBP1*, *PMEL*, *TYR*, *RPE65*), eye-field (*SIX6*, *VSX2*, *PAX6*, *OTX2*, *SIX3*, *LHX2*, *RAX*, *ZIC1*), and myogenic lineage (*MYOZ2*, *CASQ2*, *MYOD1*, *KLHL41*). **e** Table representing percentage of contribution from each sample to each of the three clusters identified in **c**.

For this purpose, we compared cultures that where either replated as described above or enriched with a combination of CD140b as a positive selection and negative selection using GD2 or CD184 at day 30 (Supplementary Fig. 4a). Replated and sorted populations were analyzed following additional 30 days culture on hrLN-521 as indicated (Fig. 4a). Single-cell analysis revealed that neither cells from the replated or sorted populations expressed pluripotency transcripts *POU5F1*, *NANOG*, *LIN28A*, *or SALL4*, but instead most robustly expressed transcripts associated with RPE (*MITF*, *CRALBP, PMEL*, *TYR*, *RPE65*, *BEST-1*) (Fig. 4b). tSNE cluster analysis of all three differentiated samples revealed three distinct clusters (Fig. 4c and Supplementary Fig. 4b). The three clusters expressed gene signatures associated with mature RPE, early eye-field progenitors, and mesodermal lineage, respectively (Fig. 4d). Distribution of the cultures using replated or prospective isolation with our markers showed that the replated cells preferentially contained a 1.2% mesoderm contaminant, although the functional significance of such low degree of impurity is questionable. The replated cells also harbored 11.3% of eye-field progenitors in contrast to just under 3% in the cultures after cell enrichment (Fig. 4d, e). Finally, signatures for several cell types present in the human retina, including bipolars, amacrines, ganglion cells, photoreceptors, lens, endothelial cells, and pan-neurons, were evaluated and no distinct clustering was found for any of the cell types (Supplementary Fig. 4c).

**Fig. 5:**
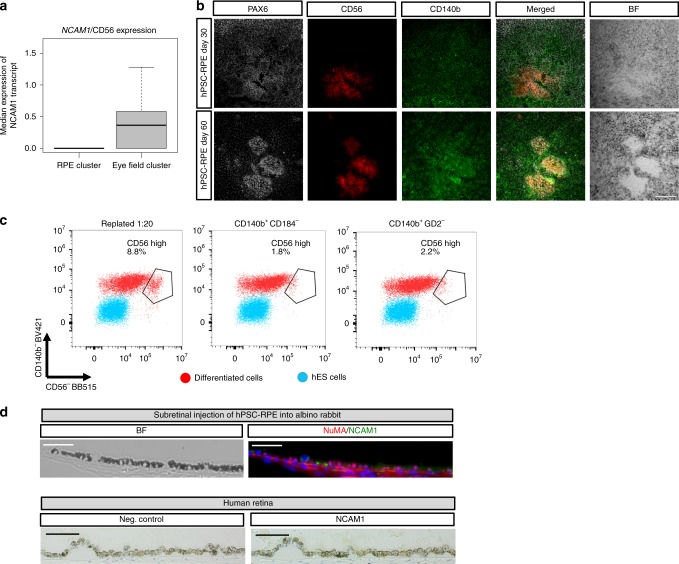
CD56 as a potential surface marker for immature hPSC-RPE. **a** Box plot comparing *NCAM1*/CD56 median expression between the RPE and eye-field clusters measured by the 10x Genomics single-cell RNA-sequencing experiment described in Fig. 4. Eye-field cluster: Median = 0.36, minimum =  0.0, maximum = 1.66, interquartile range = 0.58; RPE cluster: Median = 0.0, minimum = 0.0, maximum = 1.38, interquartile range = 0.0. **b** Bright field and immunofluorescent images showing the co-expression of the retinal progenitor marker PAX6 together with extracellular markers CD140b and CD56 in hPSC-RPE after 60 days of differentiation. **c** Flow cytometry dot plots comparing percentage of cells expressing high levels of CD56 cell surface protein at day 60 of differentiation in cells that were either replated or enriched for CD140b^+^/CD184^−^ or CD140b^+^/GD2^−^ at the 30-day replating step. **d** Upper: Bright field and immunofluorescence pictures displaying the absence of CD56 (NCAM1) expression in albino rabbit retinas injected with pigmented hPSC-RPE cells. Lower: Bright field immunohistochemistry pictures showing the absence of CD56 (NCAM1) in a human subretinal tissue section. Subretinal hPSC-RPE cells in albino rabbit were stained for human-specific NuMa antigen to distinguish them from native RPE cells. Scale bars: **b** = 100 μm; **d** = 50 μm.

Interestingly, while examining the single-cell RNA-sequencing cluster that contained genes associated with eye-field progenitors, we found that *NCAM1* also known as CD56 was enriched in this fraction of cells compared to the more mature RPE cluster (Fig. 5a). Staining of the replated cultures showed small clusters with higher CD56 intensity, which also coincided with low pigmentation levels and high levels of PAX6 protein, consistent with a less mature hPSC-RPE subpopulation (Fig. 5b). In line with the sequencing data, we also detected a larger CD56 high-expressing population in the replated cultures compared to the sorted ones (Fig. 5c). Furthermore, *NCAM1*/CD56 was not detected in mature cells following integration in the rabbit subretina, or in the naive adult human RPE, thus supporting that *NCAM1*/CD56 is lost in late-stage RPE cells (Fig. 5d).

### Markers show reproducible differentiation in multiple hPSCs

**Fig. 6:**
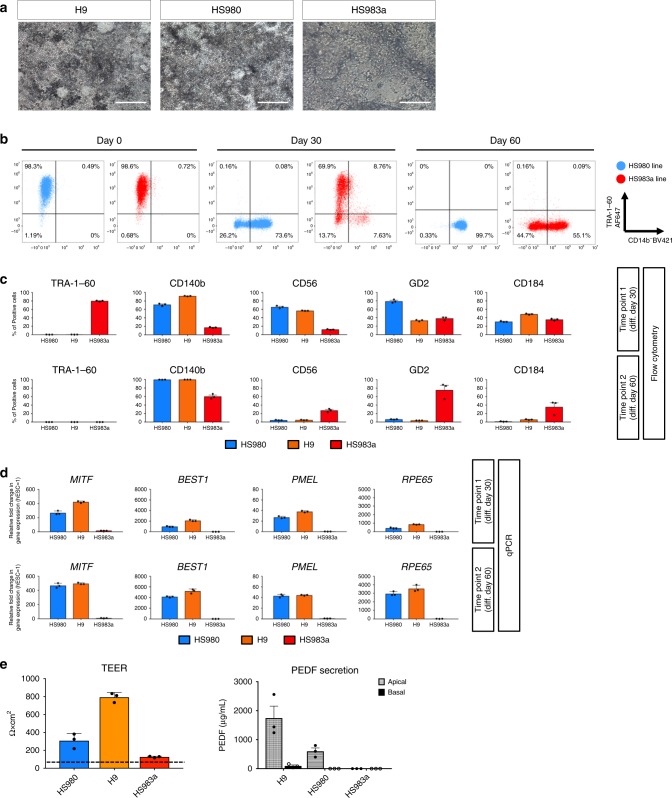
Evaluating line to line variation of the monolayer differentiation protocol using the identified cell surface markers. **a** Bright field pictures of H9, HS980, and HS983a embryonic pluripotent stem cell lines after 60 days of differentiation. **b** Illustrative dot plots showing TRA-1-60 and CD140b expression intensity measured by flow cytometry at day 0, day 30, and day 60 of differentiation in two of the three embryonic stem cell lines tested (HS980 and HS983a). **c** Percentage of positive cells for TRA-1-60, CD140b, CD56, CD184, and GD2 measured by flow cytometry at days 30 and 60 of differentiation using three different embryonic stem cell lines. **d** Gene expression analysis of RPE genes at day 30 and day 60 of differentiation. Values are normalized to *GAPDH* and displayed as relative to undifferentiated hESC. Bars represent means ± SEM from three independent experiments. **e** Functional assays demonstrating monolayer integrity measured by transepithelial resistance (TEER), and pigment epithelium-derived factor (PEDF) polarized secretion measured by ELISA. Basal values for HS983a were not detected. The TEER value for undifferentiated hESC is shown for comparison (dashed line). Bars in all bar graphs represent means ± SEM from three independent experiments. Scale bars: **a** = 200 μm. Source data are provided as a Source Data file.

A general problem with in vitro differentiation protocols is the requirement of optimization for individual cell lines. This has also been challenging for our previous suspension EB-based differentiation protocol. Strong line to line variation but also striking differences between batch to batch were noticed even within the same cell line, making robust production very challenging. We therefore tested three hESC lines (HS980, H9, and HS983a) and four hiPSC lines (CTRL-7-II, CTRL-9-II, CTRL-12-I, and CTRL-14-II) with our monolayer differentiation protocol. Following 30 days of differentiation, two (HS980 and H9) out of the three hESC lines and all four hiPSC lines tested were 60–90% CD140b^+^ and >20% positive for CD56, indicating robust induction of early RPE (Fig. 6a–c and Supplementary Fig. 5a–c). In contrast, the HS983a line failed to reach >20% positive cells for either differentiation marker at 30 days, indicating poor differentiation. In line with this observation, the same line was found to still express the pluripotency marker TRA-1-60 in 80% of the cells. Thirty days following replating, the two performing hESC lines and all four hiPSC lines had reached close to 100% CD140b levels, and had now reduced the progenitor marker CD56 to levels below 20% and the fraction of cells positive for GD2 and CD184, correlating with our previous sequencing results (Fig. 6a–c and Supplementary Fig. 5a–c). At this stage, the poor responding line had lost all significant TRA-1-60 expression and gained some signs of retinal differentiation with modest CD140b and CD56 levels while still expressing significant levels of GD2 and CD184, therefore indicating that the line can differentiate in this setting, but with significantly reduced kinetics. These results were confirmed further with transcriptional analysis of RPE-associated genes, such as *MITF*, *BEST-1*, *PMEL*, and *RPE65* (Fig. 6d and Supplementary Fig. 5d). In accordance with the antibody screen, which suggested that CD104 may be a distinctive cell surface marker labeling the mature but not the emerging immature hPSC-RPE cells (Fig. 1a, b), we could detect robust labeling in all cell lines, except HS983a, which did not show good differentiation kinetics (Supplementary Fig. 6).

As six out of seven lines differentiated well with similar kinetics, we conclude that the monolayer differentiation protocol is reproducible in a majority of hPSC lines. These data also illustrate the utility of our identified markers as quality control during the production to eliminate batch-to-batch variability or to eliminate hPSC lines that might be resistant to differentiation.

### CD140b^+^CD184^−^ enrichment can facilitate differentiation

**Fig. 7:**
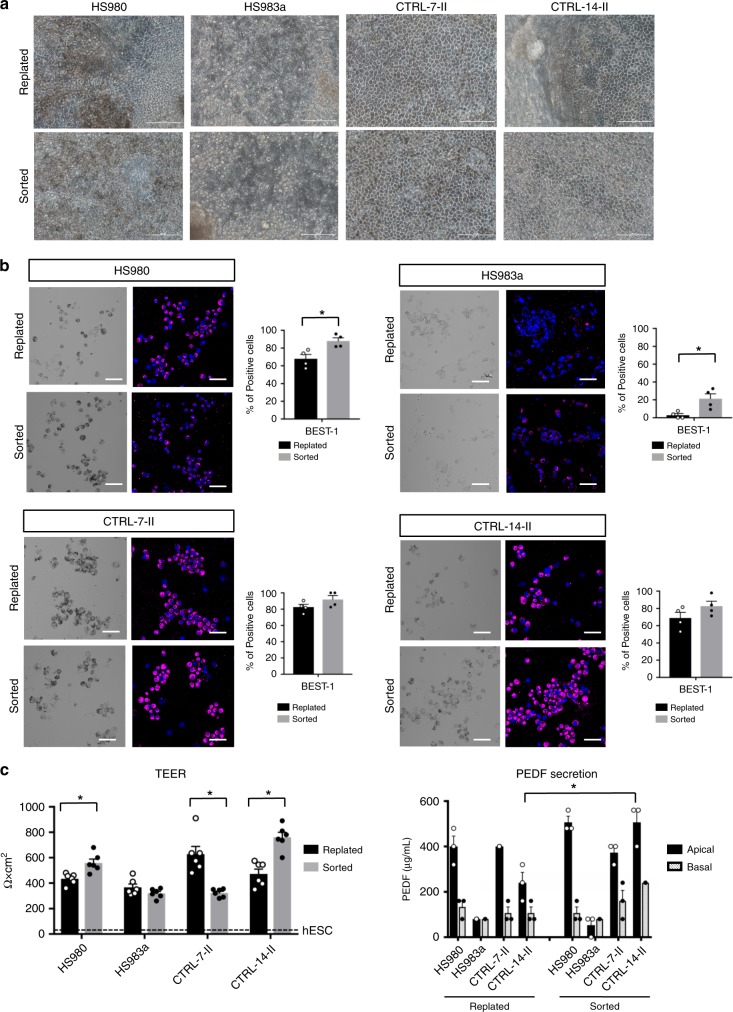
CD140b^**+**^CD184^−^ enrichment improves suboptimal hPSC-RPE differentiations. **a** Representative bright field pictures of HS980 and HS983a hESC lines and CTRL-7-II and CTRL-14-II hiPSC at day 60 of differentiation after being sorted for CD140b^+^/CD184^−^ or replated 1:20 at day 30. **b** Bright field and immunofluorescent images showing pigmentation and BEST-1 expression on cytospin mounts of day 60 hPSC-RPE that were sorted for CD140b^+^/CD184^−^ or replated 1:20 at day 30. Bar graphs compares the percentage of cells that are BEST-1^+^, manually quantified from four replicated cytospin mounts between sorted and replated hPSC-RPE in each of the four lines tested. Bars represent means ± SEM from four different images. **c** Functional assays demonstrating monolayer integrity measured by transepithelial resistance (TEER), and pigment epithelium-derived factor (PEDF) polarized secretion measured by ELISA after 60 days of differentiation in the four hPSC-RPE lines sorted for CD140b^+^/CD184^−^ or replated 1:20 at day 30. The TEER value for undifferentiated hESC is shown for comparison (dashed line). Bars represent means ± SEM from six (TEER) and three (PEDF secretion) independent experiments. (*) Asterisks represent significance with a *P* value = 0.0015 (**b**, HS980); =0.0042 (**b** HS983a); =0.046 (**c**, TEER, HS980); <0.0001 (**c** TEER, CTRL-7-II, and CTRL-14-II); <0.0001 (**c** TEER, CTRL-14-II). Scale bars: **a** = 100 μm; **b** = 50 μm. Source data are provided as a Source Data file.

We have previously shown that CD140b^+^/CD184^−^ sorting reduces the 1.2% mesoderm contaminant and the 10% eye-field progenitor cells. We therefore explored if a further improvement in differentiation and RPE function could be achieved through additional antibody-based sorting. Side-by-side comparison of replating and antibody sorting using CD140b^+^/CD184^−^ suggested that sorting can in some cases improve pigmentation and maturation, as shown by bright field pictures and BEST-1 staining (Fig. 7a, b and Supplementary Fig. 7a). The sorting step did generate BEST-1^+^ cells although still unpigmented from the poorly performing HS983a line, which otherwise showed no signs of proper RPE differentiation. Further functional assessment by measuring TEER and PEDF secretion also supported that sorting can have a positive effect (Fig. 7c), although it did not rescue the severely impaired differentiation of HS983a. The positive effect was also seen at later stages of maturation, that is, at day 90 of differentiation (Supplementary Fig. 7b). Of note, the best performing cell line CRTL-7 in this experiment showed no significant improvement by sorting, suggesting that there is no additive benefit if the cell line has already differentiated efficiently.

## Discussion

We present here the results of a comprehensive cell surface antibody screen identifying positive and negative markers for RPE cells. These markers can be used to enrich for RPE cells during and after differentiation, as well as quantitative measures to track in vitro differentiation. Taking advantage of these markers, we have established a robust and direct differentiation methodology, which will facilitate large-scale manufacturing of hPSC-RPE cells. The key to any pluripotent stem cell-based cellular replacement therapy is purity of the final product. From a safety perspective, it is critical to ensure that there are no remaining pluripotent stem cells, which could give rise to teratoma formation. Several strategies can be taken to reduce this risk. One is to negatively select for the undifferentiated cells and positively select for the cell type of interest. Although intensive efforts are in place towards pluripotent stem cell-based treatments for AMD, there has been a lack of useful cell surface markers for the RPE lineage. A previous study took an image-based strategy to identify such markers and suggested that CD59 would be useful^18^. However, we found that although RPE cells are positive for CD59, both undifferentiated and partially differentiated cells also express CD59, suggesting it less suitable for this purpose (Fig. 1a, b and Supplementary Fig. 1c). We identified that CD140b (*PDGFRB*) is specifically expressed on the RPE as they emerge during in vitro differentiations in several independent hPSC lines and can prospectively identify RPE cells from alternative cell types. CD140b/*PDGFRB* has important roles in the regulation of many biological processes, including embryonic development, angiogenesis, cell proliferation, and differentiation, thus appearing highly expressed in several tissues and cell types, such as vascular cells, decidual cells, or fibroblasts. A function of PDGFRB signaling has not been described in RPE cells. The classical function of this signaling pathway is maturation of PDGFRB expressing pericytes by PDGFB ligand secretion from the endothelial cells of the blood vessel wall^27^. It would be interesting to explore if PDGFB secreted by the choroidal vasculature also signals to the overlaying RPE cells. As for most cell surface markers, CD140b is not uniquely expressed on RPE cells, but we show that it is specific in the setting of in vitro differentiation of pluripotent stem cells as the undifferentiated cells are negative for CD140b. Importantly, we further validated that CD140b is expressed in endogenous RPE cells of the retina, and interestingly, it was found to be expressed in a polarized and apical manner. Further studies are merited to explore the RPE-specific function of PDGFRB during differentiation and RPE physiology.

Another strategy to reduce the risk of lingering pluripotent cells is to establish a differentiation protocol, which is efficient enough to eliminate all undifferentiated cells. Generally, this is achieved with most differentiation strategies towards RPE cells as current protocols spans over several weeks, efficiently eliminating undifferentiated cells. However, it is also important to generate a pure RPE product, which does not contain alternative differentiated cell types. Previous studies have evaluated purity by combined image analysis of pigmentation together with staining for several intracellular markers^28^. However, global transcriptional analysis has revealed that contaminants of cells with alternative fate can be found, such as lense-like cells expressing genes encoding crystallins in <10% of otherwise apparently homogeneous cultures^29^. Such contaminants may be difficult to identify by image analysis for pigmentation and intracellular staining of RPE markers. Flow cytometric analysis offers a good quantitative compliment to such analysis and our combined analysis of CD140b together with TRA-1-60 suggests that there are no lingering pluripotent cells. In addition, unbiased single-cell RNA-sequencing analysis is very powerful at identifying known and unknown impurities. In agreement with the flow cytometry, our single-cell global transcriptional analysis of more than 2000 hPSC-RPE did not identify any cells with transcriptional properties of undifferentiated hESC. Indeed, the vast majority of the hPSC-RPE cells expressed robust transcriptional profiles of RPE cells, whereas ~10% of the cells resembled eye-field progenitors with expression of *SIX3*/*6*, *PAX6*, *LHX2*, and *OTX2*. Further analysis would be of interest to evaluate if such eye-field progenitors would be beneficial or negative for functional integration following subretinal transplantation. The transcriptional data showed elevated transcriptional levels of *NCAM1* in this progenitor population, which was supported by positive staining in cells with low to absent pigmentation, indicating that *NCAM1*/CD56 may be a good marker to combine with CD140b to evaluate presence of eye-field progenitors or immature RPE cells. In addition, 1% of the cells expressed genes associated with mesoderm lineage, such as *MYOD1*. Clearly, mesoderm lineage is not beneficial, but it is not likely that such low fraction of mesoderm lineage would have functional consequences. However, it illustrates the power of single-cell RNA-sequencing to identify impurities in an unbiased manner. Combined positive selection for CD140b together with negative selection for either CD184 or GD2 efficiently eliminated the mesoderm contamination and reduced the fraction of eye-field progenitors, suggesting that a sorting step could be implemented to achieve a more homogeneous cell product.

Development of a clinically compliant manufacturing protocol allowing large-scale production and banking of hPSC-derived RPE cells relies on several aspects, such as xeno-free and defined components, reproducibility, streamlined process, and high production yields. Clinically compliant culture media NutriStem hPSC XF and hrLN-521 has been shown to support both hPSC and hPSC-RPE growth and expansion^10,30^. In this study, we extend on this knowledge to show that biologically relevant hrLN-111 and hrLN-521 also efficiently support direct monolayer differentiation from hPSCs to RPE with the same media, with only the addition of Activin A. The role of Activin A in RPE differentiation is in line with previous reports^6,7,25,26,31–33^. The combination of using a basal culture media together with culturing on hrLN-111 or hrLN-521 proves to be very robust and translatable to multiple lines. The elimination of EB differentiation, which involved manual dissection of pigmented areas, makes the protocol significantly more streamlined and amenable to automatization in closed systems as it only requires media changes and one bulk passage. One striking benefit of changing to monolayer differentiation on laminins is the increased yield, with 1300-fold expansion (1:20 dilution) from starting hPSC material into fully functional hPSC-RPE. Considering that current cell replacement approaches for treating AMD use a dose of 100,000–200,000 cells per eye^34,35^, our protocol could generate in 60 days cells equivalent to 6500–13,000 treatment doses, from a starting culture of only million undifferentiated hPSCs.

In conclusion, we have identified several cell surface markers, including CD140b, CD56, CD104, CD184, and GD2, that together can be used as quantitative quality-control assays to evaluate maturation and purity of hPSC-RPE differentiation, as well as for positive and negative enrichment to generate a pure RPE product. With the aid of these markers to quantify the differentiation process, we have established a xeno-free and defined, manual selection-free monolayer differentiation protocol, which is amenable to GMP-compliant manufacturing allowing large-scale production and banking of hPSC-derived RPE cells as source for cell based reparative therapy of AMD.

## Methods

### Human cell surface marker screening

hESC and hPSC-RPE cells were dissociated into single cells using TrypLE for 5–10 min. Optic vesicles were dissociated into single cells using TrypLE Select (Gibco, Invitrogen) for 10 min, followed by physical dissociation through a 20 G needle. To allow the simultaneous analysis of these different populations in the same sample, hESC, hPSC-RPE cells, and optic vesicle cells were labeled with CellTrace™ CFSE (0.25 µM) for 7 min at 37 °C or CellTrace™ Violet (5 µM) for 20 min at 37 °C following the manufacturer’s protocol (Thermo Fisher Scientific). Then, the three cell types were stained using the BD Lyoplate™ Screening Panels (BD Biosciences) following the manufacturer’s protocol. The barcoding of cells allowed to distinguish easily between the three groups of cells and to minimize sample variability during the screening. Samples were analyzed on 96-well plates on a LSRFortessa equipped with 405, 640, 488, 355, and 561 nm lasers (BD Biosciences) or a CytoFLEX equipped with 405, 638, 488, and 561 nm lasers (Beckman Coulter). Non-viable cells were excluded from the analysis using 7-AAD (7-aminoactinomycin D) nucleic acid dye (BD Biosciences). Analysis of the data was carried out using the FlowJo v.10 software (Tree Star). The cell surface marker screen was performed once for 3D optic vesicles, and once for hPSC-RPE day 60.

### Cell culture

hESC lines HS980 and HS983 were previously derived and cultured under xeno-free and defined conditions^30^ (Swedish Ethical Review Authority: 2011/745:31/3). Donors gave their informed consent for the derivation and subsequent use of the hESC lines. The WA09/H9 hESC line was obtained from Wicell and was adapted to feeder-free culture on hrLN-521 (10 μg/mL, Biolamina). Cells were maintained by clonal propagation on hrLN-521-coated plates in NutriStem hPSC XF medium (Biological Industries), in a 5% CO_2_/5% O_2_ incubator, and passaged enzymatically at 1:10 ratio every 5–6 days.

hiPSC lines CTRL-7-II, CTRL-9-II, CTRL-12-I, and CTRL-14-II were kindly provided by the Karolinska Institutet iPSC Core facility (Swedish Ethical Review Authority: 2012/208-31/3, 2010/1778-31/4). Donors gave their informed consent for the derivation and subsequent use of the hiPSC lines. Cells were maintained by clonal propagation on hrLN-521-coated plates (Biolamina) in NutriStem hPSC XF medium (Biological Industries), in a 5% CO_2_/5% O_2_ incubator and passaged enzymatically at 1:10 ratio every 5–6 days.

For passaging, confluent cultures were washed twice with phosphate-buffered saline (PBS) without Ca^2+^ and Mg^2+^ and incubated for 5 min at 37 °C, 5% CO_2_/5% O_2_ with TrypLE Select. The enzyme was then carefully removed and cells were collected in fresh pre-warmed NutriStem hPSC XF medium by gentle pipetting to obtain a single-cell suspension. Cells were centrifuged at 300 × *g* for 4 min, the pellet resuspended in fresh pre-warmed NutriStem hPSC XF medium, and cells plated on a freshly hrLN-521-coated dish. Two days after passage, the medium was replaced with fresh pre-warmed NutriStem hPSC XF medium and changed daily.

### hPSC-RPE monolayer differentiation

A step-by-step protocol describing the differentiation protocol can be found at Nature Protocol Exchange^36^. hESC or hiPSC were plated at a cell density of 2.4 × 10^4^ cells/cm^2^ on laminin-coated dishes (20 μg/mL) using NutriStem hPSC XF medium. Rho-kinase inhibitor (Y-27632, Millipore) at a concentration of 10 μM was added during the first 24 h, while cells were kept at 37 °C, 5% CO_2_/5% O_2_. After 24 h, hPSC medium was replaced with differentiation medium NutriStem hPSC XF without basic fibroblast growth factor (bFGF) and transforming growth factor-β (TGFβ) (Biological Industries) and cells were placed at 37 °C, 5% CO_2_/21%O_2_. From day 6 after plating, 100 ng/mL of Activin A (R&D Systems) was added to the media. Cells were fed three times a week and kept for 30 days. Monolayers were then trypsinized using TrypLE Select (Gibco, Invitrogen) for 10 min at 37 °C, 5% CO_2_. The enzyme was carefully removed and the cells were collected in fresh pre-warmed NutriStem hPSC XF medium without bFGF and TGFβ by gentle pipetting to obtain a single-cell suspension. The cells were centrifuged at 300 × *g* for 4 min, the pellet was resuspended, passed through a cell strainer (ø 40 μm, BD Biosciences), and cells were seeded on laminin-coated dishes (hrLN-111 and hrLN-521 at 20 mg/mL) at different cell densities ranging from 1.4 × 10^6^ to 1.4 × 10^4^ cells/cm^2^. Replated cells were fed three times a week during the subsequent 30 days with NutriStem hPSC XF medium without bFGF and TGFβ. For hPSC-RPE in vitro differentiation in 3D suspension EBs, we followed our previously published protocol^10^. Briefly, pluripotent stem cells were cultured to confluence on rhLN-521 and manually scraped to produce EBs using a 1000 μL pipette tip. The EBs were then cultured in suspension in low attachment plates (Corning) at a density of 5–7 × 10^4^ cells/cm^2^. Differentiation was performed in custom-made NutriStem hESC XF medium in which bFGF and TGFβ have been eliminated with media change twice a week. Ten micromoles of Rho-kinase inhibitor (Y-27632, Millipore) was added to the suspension cultures only during the first 24 h. Following 5 weeks differentiation, pigmented areas were mechanically cut out of the EBs using a scalpel. Cells were then dissociated using TrypLE Select, followed by flushing through a 20 G needle and syringe. Cells were seeded through a cell strainer (ø 40 μm, BD Biosciences) on LN-coated dishes at a cell density of 0.6–1.2 × 10^4^ cells/cm^2^ and fed twice a week with the same differentiation medium referred above. Bright field images were acquired with a Nikon Eclipse TE2000-S microscope and a Canon SX170 IS camera was used to capture pigmentation from the top of the wells.

### Quantitative real-time PCR

Total RNA was isolated using the RNeasy Plus Mini Kit and treated with RNase-free DNase (both from Qiagen). Complementary DNA (cDNA) was synthesized using 1 μg of total RNA in 20 μL reaction mixture, containing random hexamers and Superscript III reverse transcriptase (Gibco, Invitrogen), according to the manufacturer’s instructions.

*Taq* polymerase together with Taqman probes (Thermo Fisher Scientific) for *GAPDH* (cat. no. 4333764F), *NANOG* (cat. no. Hs02387400_g1), *POU5F1* (cat. no. Hs03005111_g1), *MITF* (cat no. Hs01117294_m1), *BEST-1* (cat. no. Hs00188249_m1), *RPE65* (cat. no. Hs01071462_m1), *TYR* (cat. no. Hs00165976_m1), *PMEL* (cat. no. Hs00173854_m1), *MAP2* (cat no. Hs00258900_m1), *PDGFRB* (cat no. Hs01019589_m1), and *TUBB3* (cat no. Hs00801390_s1) were used. Samples were subjected to real-time PCR amplification protocol on StepOne^TM^ real-time PCR System (Applied Biosystems). Three independent experiments were performed for every condition and technical duplicates were carried for each reaction. Results are presented as mean ± SEM (standard error of the mean).

### Flow cytometry

hPSC-RPE growing on the tested substrates were dissociated into single cells using TrypLE Select. Samples were stained with BV421 Mouse Anti-Human CD140b- (BD Biosciences 564124, clone [28D4], 10 μg/mL), PE Mouse Anti-Human CD140b- (BD Biosciences 558821, clone [28D4], 10 μg/mL), BB515 Mouse Anti-Human CD56- (BD Biosciences 564489, clone [B159], 2.5 μg/mL), Alexa Fluor 647 Mouse Anti-Human TRA-1-60- (BD Biosciences 560850, clone [TRA-1-60], 0.6 μg/mL), BV421 Mouse Anti-Human CD184- (BD Biosciences 562448, clone [12G5], 2.5 μg/mL), BV421 Mouse Anti-Human Disialoganglioside GD2- (BD Biosciences 564223, clone [14.G2a], 2.5 μg/mL), PECy7 Mouse Anti-Human CD184- (BD Biosciences 560669, clone [12G5], 2.5 μg/mL), BV605 Mouse Anti-Human Disialoganglioside GD2- (BD Biosciences 744071, clone [14.G2a], 2.5 μg/mL), and BV605 Rat Anti-Human CD104- (BD Biosciences 744152, clone [439-9B], 2 μg/mL) conjugated antibodies (Supplementary Data 2), diluted in 2% fetal bovine serum (FBS, Thermo Fisher Scientific) and 1 mM EDTA (Sigma). Cells were incubated with the conjugated antibodies on ice for 30 min. Fluorescence minus one (FMO) controls were included for each condition to identify and gate-negative and gate-positive cells. Stained cells were analyzed using a CytoFLEX flow cytometer equipped with 488, 561, 405, and 640 nm lasers (Beckman Coulter). Analysis of the data was carried out using the FlowJo v.10 software (Tree Star).

Cell sorting was performed on hPSC-RPE cultures after 21 or 30 days of differentiation. Cells were incubated with the mentioned conjugated antibodies on ice for 30 min. FMO controls were included for each condition to identify and gate-negative and -positive cells. Stained cells were then sorted using a BD FACS Aria Fusion Cell Sorter (BD Biosciences) using the FACSDiva Sofware v8.0.1.

Right after sorting, 70,000 cells diluted in 100 μL of 2% FBS and 1 mM EDTA (Sigma) were cytospinned for 5 min at 400 r.p.m. onto glass slides. Slides were left to dry overnight at room temperature, followed by fixing with 4% methanol-free formaldehyde at room temperature for 10 min and immunofluorescence staining.

### Immunofluorescence

Protein expression of day 60 hPSC-RPE monolayers was assessed with immunofluorescence. Cells were fixed with 4% methanol-free formaldehyde at room temperature for 10 min, followed by permeabilization with 0.3% Triton X-100 (Sigma) in Dulbecco’s PBS (D-PBS) for 10 min and blocking with 4% FBS and 0.1% Tween-20 (Sigma) in D-PBS for 1 h. Primary antibodies were diluted to the specified concentrations in 4% FBS, 0.1% Tween-20, D-PBS solution: PAX6 (1:400, BioLegend 901301), NANOG (1:200, ReproCell RCAB003P), BEST-1 (1:100, Millipore MAB5466), MITF (1:200, Abcam ab3201, clone [D5]), ZO-1 (zonula occludens-1, 1:100, Invitrogen 40-2200), CRALBP (1:250, Abcam ab15051, clone [B2]), PDGFRB (CD140b) (1:100, BD Biosciences 558820, clone [28D4]), CD56 (1:100, BD Biosciences 555513, clone [B159]), CXCR4 (CD184) (1:300, Abcam ab1670), and ganglioside GD2 (1:200, Santa Cruz Biotechnology sc-53831, clone [14G2a]) (Supplementary Data 2). The primary antibodies were incubated overnight at 4 °C, followed by 2 h incubation at room temperature with secondary antibodies: donkey anti-mouse IgG (H + L) Alexa Fluor 488, donkey anti-mouse IgG Alexa Fluor (H + L) 555, donkey anti-mouse IgG Alexa Fluor (H + L) 647, donkey anti-rabbit IgG (H + L) Alexa Fluor 647, goat anti-mouse IgG1 Alexa Fluor 568, and goat anti-mouse IgG2a Alexa Fluor 488 (all of them from Thermo Fisher Scientific, A21202, A31570, A31571, A31573, A21124, A21131, respectively) diluted 1:1000 in 4% FBS, 0.1% Tween-20, and D-PBS solution. Nuclei were stained with Hoechst 33342 (1:1000, Invitrogen H3570) (Supplementary Data 2). Images were acquired with Zeiss LSM710-NLO point scanning confocal microscope. Post-acquisition analysis of the pictures was performed using Imaris (Bitplane) and/or ImageJ software.

### Histology and tissue immunostaining

Immediately after euthanasia by intravenous injection of 100 mg/kg pentobarbital (Allfatal vet. 100 mg/mL, Omnidea), the eyes were enucleated and the bleb injection area marked with green Tissue Marking Dye (TMD) (Histolab Products). An intravitreal injection of 100 μL fixing solution (FS) consisting of 4% buffered formaldehyde (Solvenco AB) was performed before fixation in FS for 24–48 h, and embedding in paraffin. Four-micrometer serial sections were produced through the TMD-labeled area and every four sections were stained with hematoxylin–eosin.

For immunostaining, slides were deparaffinized in xylene, dehydrated in graded alcohols, and rinsed with ddH_2_O and Tris-buffered Saline (TBS, pH 7.6). Antigen retrieval was achieved in 10 mM citrate buffer (trisodium citrate dihydrate, Sigma-Aldrich, pH 6.0) with 1:2000 Tween-20 (Sigma-Aldrich) at 96 °C for 30 min, followed by 30 min cooling at room temperature. Slides were washed with TBS and blocked for 30 min with 10% normal donkey serum (Abcam) diluted in TBS containing 5% (w/v) IgG and protease-free bovine serum albumin (Jackson Immunoresearch) in a humidified chamber. Primary antibodies diluted in blocking buffer were incubated overnight at 4 °C: human nuclear mitotic apparatus protein (NuMA) (1:200, Abcam ab84680), BEST-1 (1:200, Millipore MAB5466), CD140b/PDGFRB (1:100, Santa Cruz Biotechnology sc-432), and CD56/NCAM1 (1:100, Santa Cruz Biotechnology sc-7326, clone [123C3]) (Supplementary Data 2). Secondary antibodies (donkey anti-rabbit IgG (H + L) Alexa Fluor 555 A31572 and donkey anti-mouse IgG (H + L) Alexa Fluor 647 A31571, both from Thermo Fisher Scientific) (Supplementary Data 2) diluted 1:200 in blocking buffer were incubated 1 h at room temperature. Sections were mounted with vector Vectashield with DAPI ((4′,6-diamidino-2-phenylindole)) mounting medium (Vector Laboratories) under a 24 × 50 mm^2^ coverslip.

For immunohistochemistry, slides were deparaffinized followed by antigen retrieval (ER2 solution, pH 9, 20 min, Leica Biosystems) and staining (IHC Protocol F) for CD140b/PDGFRB (1:100, Santa Cruz Biotechnology sc-432) and CD56/NCAM1 (1:100, Santa Cruz Biotechnology sc-7326, clone [123C3]) antibodies (Supplementary Data 2) on Bond RXm instrument (Leica Biosystems).

Images were taken with Olympus IX81 fluorescence inverted microscope or Zeiss LSM710-NLO point scanning confocal microscope. Post-acquisition analysis of the pictures was performed using the ImageJ software.

### Phagocytosis assay

Fluorescein isothiocyanate-labeled bovine POSs were isolated and kindly given by Dr. E.F. Nandrot from Institut de la Vision, Paris^37^. hPSC-RPE cells were cultured on transwell membrane (0.33 cm^2^, Corning) coated with hrLN-521 20 μg/mL for 1 month after seeding. Cells were incubated at 37 °C or 4 °C for 16 h with 2.42 × 10^6^ thawed POS/Transwell diluted in DMEM (Dulbecco’s modified Eagle’s medium) or CO_2_-independent media (both from Thermo Fisher Scientific), respectively. After incubation, cells were quenched with Trypan Blue Solution 0.2% (Gibco, Invitrogen) for 10 min at room temperature, fixed with 4% methanol-free formaldehyde (Polysciences) at room temperature for 10 min, and permeabilized with 0.3% Triton X-100 in D-PBS for 15 min. Rhodamine phalloidin staining (1:1000, 20 min at room temperature, Biotinum 00027) (Supplementary Data 2) was used to visualize the cell boundaries. Nuclei were stained with Hoechst 33342 (1:1000, 20 min at room temperature, Invitrogen).

Images were acquired with Zeiss LSM710-NLO point scanning confocal microscope. Post-acquisition analysis of the pictures was performed using Imaris (Bitplane) and POS quantifications were done with the CellProfiler 2.1.1 software. Modules used: LoadImages, ColorToGrey, IdentifyPrimaryObjects, MeasureObjectSizeShape, SaveImages, and ExportToSpreadsheet. Objects were identified by a typical diameter of 10–40 pixel units using Two Classes, Global, Otsu, Weighted variance thresholding method with 0.01 and 1.0 lower and upper bounds, and 2.1 correction factor, with clumped objects distinguished by intensity.

### Enzyme-linked immunosorbent assay

hPSC-RPE cells were cultured on Transwell membranes (0.33 cm^2^, Millipore) coated with different substrates. Supernatants from both the hPSC-RPE apical and basal sides (meaning upper and lower compartments of the transwell, respectively) were collected 60 h after the medium was changed. PEDF secretion levels were measured in triplicates for each condition with commercially available human PEDF ELISA Kits (BioVendor RD191114200R) were used, in accordance with the manufacturer’s instructions, after 60 days of culture. The optical density Sreadings were measured using SpectraMax 250 Microplate Reader (Molecular Devices). Results are presented as mean ± SEM.

### TEER measurements

Transepithelial electrical resistance RPE cells plated on Transwells (0.33 cm^2^, Millipore) were measured using the Millicell Electrical Resistance System volt-ohm meter (Millicell ERS-2, Millipore), according to the manufacturer’s instructions. Sixty-day cultures were equilibrated outside the incubator at room temperature for 15–20 min before the experiment. Measurements were performed in unchanged culture media in triplicate for each condition, at three different positions of each well. Averages were used for further analysis. The background resistance was determined from a blank culture insert in the same media coated with the corresponding substrate but without cells, and subtracted from the respective experiment condition. Measurements are reported as resistance in ohms times the area in square centimeters (Ω × cm^2^). Results are presented as mean ± SEM.

### Scanning electron microscopy

hPSC-RPE cells were grown on transwell inserts coated with LN521 (20 μg/mL) for 60 days. They were fixed by immersion in 2.5% glutaraldehyde in 0.1 M phosphate buffer, pH 7.4. The transwell membrane was cut out and washed in MilliQ water prior to stepwise ethanol dehydration and critical-point-drying using carbon dioxide (Leica EM CPD 030). Inserts were mounted on specimen stubs using carbon adhesive tabs and sputter coated with a thin layer of platinum (Quorum Q150T ES). Scanning electron microscopy images were acquired using an Ultra 55 field emission scanning electron microscope (Zeiss, Oberkochen, Germany) at 3 kV and the SE2 detector.

### Transmission electron microscopy

hPSC-RPE cells were grown on transwell inserts coated with LN521 (20 μg/mL) for 60 days. They were fixed by immersion in 2.5% glutaraldehyde in 0.1 M phosphate buffer, pH 7.4. The transwell membrane was cut out and into thin strips, rinsed in 0.1 M phosphate buffer, followed by post fixation in 2% osmium tetroxide in 0.1 M phosphate buffer, pH 7.4, at 4 °C for 2 h. The membrane strips were subjected to stepwise ethanol dehydration and finally flat embedded in LX-112. Ultrathin sections (~50–60 nm) were prepared using a Leica EM UC7 and contrasted with uranyl acetate, followed by lead citrate. Transmission electron microscopy imaging was done on a Hitachi HT7700 transmission electron microscope (Hitachi High-Technologies) operated at 80 kV and digital images were acquired using a Veleta CCD camera (Olympus Soft Imaging Solutions).

### Single-cell RNA-sequencing bioinformatic analysis

Sixty-day hPSC-RPE cells were dissociated using TrypLE Select and passed through a cell strainer (ø 40 μm, BD Biosciences). They were resuspended at a concentration of 1000 cells/μL in 0.04% BSA in PBS. Cells were transported at 4 °C to the Eukaryotic Single Cell Genomics Facility (ESCG, SciLifeLab, Stockholm, Sweden) where a 3′ cDNA library was prepared for single-cell RNA-sequencing with the 10x Genomics platform (10x Genomics) using the NovaSeq 6000 software. Cell Ranger 2.1.1 (10x Genomics) pipeline was used to convert Illumina base call files to fastq format, align sequencing reads to the hg19 transcriptome using the STAR aligner^38^, and generate feature-barcode matrices. Cell Ranger quality-control filtered cells (718, 810, 931, and 1129 cell-containing droplets were captured for CD140b^+^GD2^−^, CD140b^+^CD184^−^, replated 1:20 and hESC samples, respectively) were analyzed in R version 3.5.1 (R Core Team)^39^, using Seurat suite version 2.3.4^40,41^. As a further quality-control measure, RPE cells with uniquely expressed genes (≥2000 to ≤5000), UMIs (≥10,000 to ≤30,000) and percentage of UMI mapping to MT genes (≥0.025 to ≤0.10) were selected. Similarly, hESC with uniquely expressed genes (≥2000 to ≤8000), UMIs (≥10,000 to ≤80,000), and percentage of UMI mapping to MT genes (≥0.025 to ≤0.10). This filtration step resulted in final dataset of 616, 725, 779, and 905 cells for CD140b^+^GD2^−^, CD140b^+^CD184^−^, replated 1:20 and hESC samples, respectively. Before dimensionality reduction by principal component (PC) analysis, cell–cell variation in gene expression driven by UMIs, mitochondrial gene expression, and cell-cycle stages were regressed out during data scaling process^42^. Variable genes within RPE samples were selected based on their normalized average expression and dispersion (expression cut-off = 0.0125–5, and bottom dispersion cut-off = 0.5). For PC selection, findings of PCHeatmap, jackStraw, PC standard deviations, and Clustree analysis were assessed^43^. The first 15 PCs were used for the tSNE projection^44^ and clustering analysis (resolution = 0.1, perplexity = 40).

Cell clusters were analyzed by two approaches. Top differential genes were first identified for each cluster using Wilcoxon’s rank-sum test. Secondary, signature gene expression (module scores) was computed for undifferentiated hESC and several cell types present in human retina. Cells expressing mesoderm markers were manually subdivided in a separate cluster using interactive plotting features of Seurat. Data is uploaded in ArrayExpress (EMBL-EBI)—see details below.

### Animals

After approval by the Northern Stockholm Animal Experimental Ethics Committee (DNR N25/14), 10 New Zealand white albino rabbits (provided by Lidköpings rabbit farm, Lidköping, Sweden) aged 5 months, weighing 3.5 to 4.0 kg were used in this study. All experiments were conducted in accordance with the Statement for the Use of Animals in Ophthalmic and Vision Research.

### Subretinal transplantation

hPSC-RPE monolayers were washed with PBS, incubated with TrypLE, and dissociated to single-cell suspension. Cells were counted in a Neubauer hemocytometer chamber using 0.4% Trypan blue (Thermo Fisher Scientific Corp.), centrifuged at 300 × *g* for 4 min, and the cell pellet was resuspended in freshly filter-sterilized PBS to a final concentration of 1000 cells/μL. The cell suspension was then aseptically aliquoted into 600 μL units and kept on ice until surgery.

Animals were put under general anesthesia by intramuscular administration of 35 mg/kg ketamine (Ketaminol, 100 mg/mL, Intervet) and 5 mg/kg xylazine (Rompun vet. 20 mg/mL, Bayer Animal Health), and the pupils were dilated with a mix of 0.75% cyclopentolate/2.5% phenylephrine (APL). Microsurgeries were performed on both eyes using a 2-port 25 G transvitreal pars plana technique (Alcon Accurus, Alcon Nordic) as described previously^45^. The cell suspension was drawn into a 1 mL syringe connected to an extension tube and a 38G polytip cannula (MedOne Surgical Inc). Without prior vitrectomy, the cannula was inserted through the upper temporal trocar. After proper tip positioning, ascertained by a focal retinal flare, 50 μL of cell suspension (equivalent to 50,000 cells) was injected slowly subretinally ~6 mm below the inferior margin of the optic nerve head, forming a uniform bleb that was clearly visible under the operating microscope. Care was taken to maintain the tip within the bleb during the injection to minimize reflux. After instrument removal, light pressure was applied to the self-sealing suture-less sclerotomies. Two micrograms (100 μL) of intravitreal triamcinolone (Triescence, Alcon Nordic) was administered 1 week prior to the surgery, and no post-surgical antibiotics were given.

### Statistics and reproducibility

For statistical analyses, two-way analysis of variance and post hoc multiple comparisons using Tukey’s test correction were performed to assess the in vitro differences of the different densities assessed and sorting versus replated conditions in TEER and PEDF secretion assays.

All quantifications were performed unblinded. Statistical parameters including the definitions and exact value of *n* (e.g., total number of experiments, replications, etc.), deviations, *P* values, and the types of the statistical tests are reported in the figures, the corresponding figure legends, and in this section. Statistical analysis was carried out using Prism 7 (GraphPad Software, version 7.0c). In all cases, statistical analysis was conducted on data from at least three biologically independent experimental replicates. Comparisons between groups were planned before statistical testing and target effect sizes were not predetermined. Error bars displayed on graphs represent the mean ± SEM of at least three independent experiments. All micrographs shown are representative images of three independent experiments, unless otherwise specified (e.g., Fig. 1c).

### Reporting summary

Further information on research design is available in the Nature Research Reporting Summary linked to this article.

## Supplementary information

Supplementary Information

Supplementary Data 1

Supplementary Data 2

Description of Additional Supplementary Files

Reporting Summary

## Data Availability

The authors declare that all data supporting the findings of this study are available within the article and its supplementary information files or from the corresponding author upon reasonable request. The raw data for the single-cell RNA-sequencing have been deposited in the ArrayExpress (EMBL-EBI) database under accession code: E-MTAB-7742. The source data underlying Fig. 1a, b, g, 2c, d, 3c–f, 6c–e, and 7b, c and Supplementary Figs. 1e, 2b–d, 5c–e, 6, and 7b are provided as a Source Data file.

## Source data

Source Data
