# Publisher Correction: Identification of cell surface markers and establishment of monolayer differentiation to retinal pigment epithelial cells

**DOI:** 10.1038/s41467-020-16166-z

**Published:** 2020-05-06

**Authors:** Alvaro Plaza Reyes, Sandra Petrus-Reurer, Sara Padrell Sánchez, Pankaj Kumar, Iyadh Douagi, Hammurabi Bartuma, Monica Aronsson, Sofie Westman, Emma Lardner, Helder André, Anna Falk, Emeline F. Nandrot, Anders Kvanta, Fredrik Lanner

**Affiliations:** 1 Department of Clinical Sciences, Intervention and Technology, Karolinska Institutet, Stockholm, Sweden; 2 Ming Wai Lau Center for Reparative Medicine, Stockholm node, Karolinska Institutet, Stockholm, Sweden; 3 Division of Obstetrics and Gynecology, Karolinska Universitetssjukhuset, Stockholm, Sweden; 4 Department of Clinical Neuroscience, Division of Eye and Vision, St. Erik Eye Hospital, Karolinska Institutet, Stockholm, Sweden; 5 Department of Medicine, Center for Hematology and Regenerative Medicine, Karolinska Institutet, Stockholm, Sweden; 6 Department of Neuroscience, Karolinska Institutet, Stockholm, Sweden; 7 INSERM, CNRS, Institut de la Vision, Sorbonne Université, 75012 Paris, France

**Keywords:** Embryonic stem cells, Stem-cell research

Correction to: *Nature Communications* 10.1038/s41467-020-15326-5, published online 30 March 2020.

The original HTML and PDF versions of this Article were updated shortly after publication because the previous HTML and PDF versions incorrectly linked to an unrelated Transparent Peer Review file.

