# Publisher Correction: Identification of cell surface markers and establishment of monolayer differentiation to retinal pigment epithelial cells

**DOI:** 10.1038/s41467-020-17365-4

**Published:** 2020-07-09

**Authors:** Alvaro Plaza Reyes, Sandra Petrus-Reurer, Sara Padrell Sánchez, Pankaj Kumar, Iyadh Douagi, Hammurabi Bartuma, Monica Aronsson, Sofie Westman, Emma Lardner, Helder André, Anna Falk, Emeline F. Nandrot, Anders Kvanta, Fredrik Lanner

**Affiliations:** 1 Department of Clinical Sciences, Intervention and Technology, Karolinska Institutet, Stockholm, Sweden; 2 Ming Wai Lau Center for Reparative Medicine, Stockholm node, Karolinska Institutet, Stockholm, Sweden; 3 Division of Obstetrics and Gynecology, Karolinska Universitetssjukhuset, Stockholm, Sweden; 4 Department of Clinical Neuroscience, Division of Eye and Vision, St. Erik Eye Hospital, Karolinska Institutet, Stockholm, Sweden; 5 Department of Medicine, Center for Hematology and Regenerative Medicine, Karolinska Institutet, Stockholm, Sweden; 6 Department of Neuroscience, Karolinska Institutet, Stockholm, Sweden; 7 INSERM, CNRS, Institut de la Vision, Sorbonne Université, 75012 Paris, France

**Keywords:** Embryonic stem cells, Stem-cell research

Correction to: *Nature Communications* 10.1038/s41467-020-15326-5, published online 30 March 2020.

The original version of this Article contained an error in the Methods section, which incorrectly read ‘A step-by-step protocol describing the differentiation protocol can be found at Nature Protocol Exchange (ref. 36)’. The correct version states ‘Protocol Exchange’ in place of ‘Nature Protocol Exchange’. This has been corrected in both the PDF and HTML versions of the Article.

The original version of this Article contained an error in ref. 36, which was incorrectly given with the wrong journal name as: ‘*Nat. Protoc.’* and incorrect DOI. The correct form of ref. 36 is:

Plaza Reyes, A. et al. Xeno-free, chemically defined and scalable protocol to produce hPSC-derived RPE monolayer. *Protoc. Exch*. 10.21203/rs.3.pex-635/v1 (2020).

This has been corrected in both the PDF and HTML versions of the Article.

