# Supplementary material for: Identification of cell surface markers and establishment of monolayer differentiation to retinal pigment epithelial cells
Source: Nat Commun. 2020 Mar 30;11:1609. doi: 10.1038/s41467-020-15326-5 (PMC7105463; doi:10.1038/s41467-020-15326-5)
Supplement: Supplementary file 1 — Supplementary Information [file 41467_2020_15326_MOESM1_ESM.pdf]

SUPPLEMENTARY INFORMATION

Identification of cell surface markers and establishment of monolayer differentiation to retinal pigment epithelial cells

Plaza Reyes et al.

Supplementary Figures 1 - 7

Supplementary Figure 1

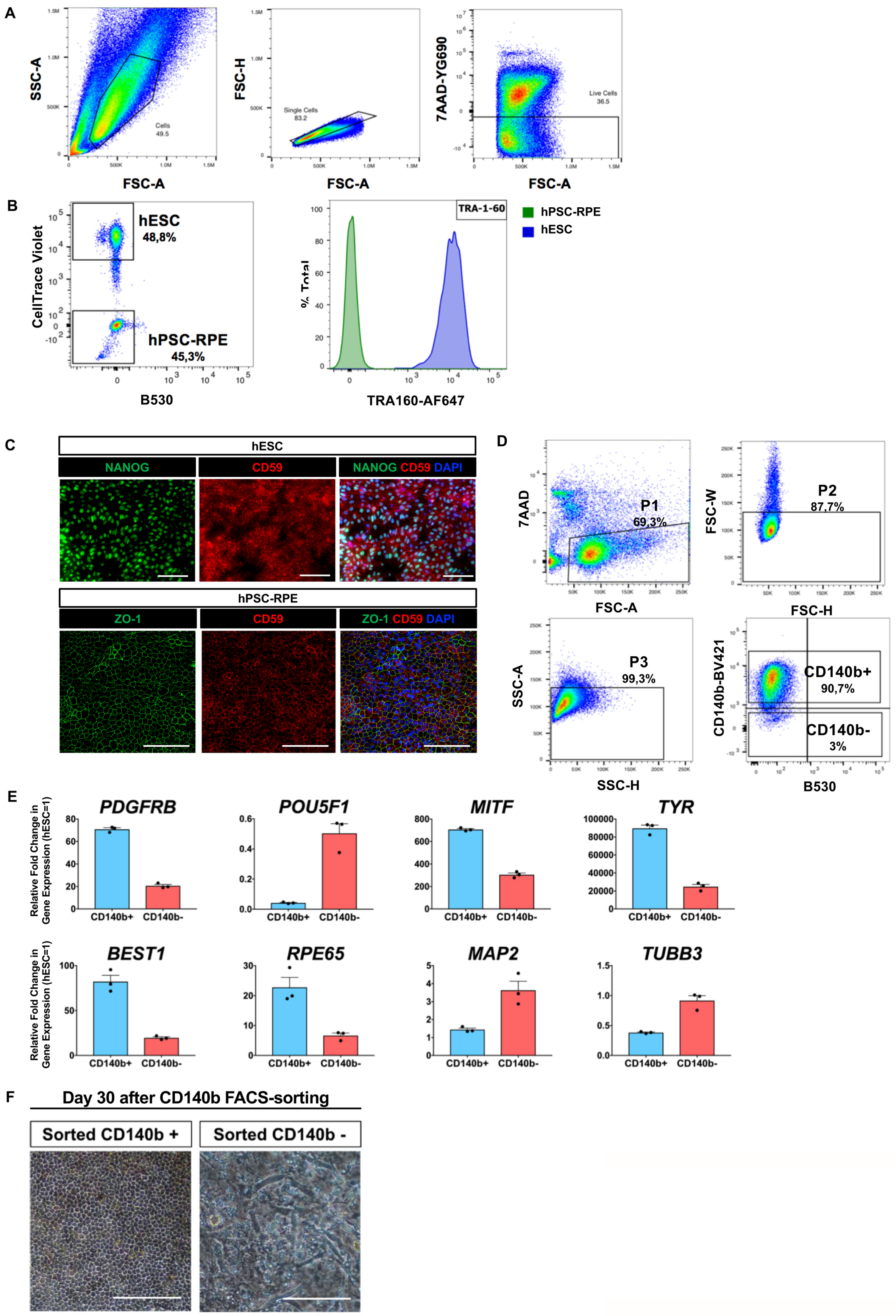

CD140b can specifically enrich hPSC-RPE cell population during differentiation.

(A) Representative flow cytometry dot plot showing general gating strategy followed to select single live cells in each sample that was analyzed in Figures1A-B. **(B)** Left: Representative flow cytometry dot plot showing gating strategy followed after barcoding hESC and hPSC-RPE for the screening against a commercial antibody panel. hESC were stained here with CellTrace Violet dye while hPSC-RPE were left unstained; gated cells were then analysed for individual surface protein expression. Such strategy allowed us to compare the expression of a certain surface protein in more than one cell type in the same sample. Right: Depictive flow cytometry histogram exemplifying expression of a certain surface protein, here TRA-1-60, in hESC and hPSC-RPE cells after discriminating each cell type with the barcoding strategy. **(C)** Immunofluorescence image showing CD59 and NANOG co-expression in undifferentiated hESCs, and CD59 and ZO-1 co-expression in hPSC-RPE cells. **(D)** Gating strategy used for isolation of cell populations profiled in Figures 1F-G. **(E)** Gene expression analysis of pluripotency, RPE and neuronal-specific genes in the CD140b+ and CD140b- sorted populations. Values are normalized to *GAPDH* and displayed as relative to undifferentiated hESC. Bars represent means±SEM from three independent experiments. **(F)** Bright field pictures of cell cultures on hrLN-521 30 days after FACS sorting hPSC-RPE differentiations for CD140b. CD140b+ population shown on the left and CD140b- population shown on the right. Scale bars: (C) and (F) = 100 µm. Source data are provided as a Source Data file.

Supplementary Figure 2

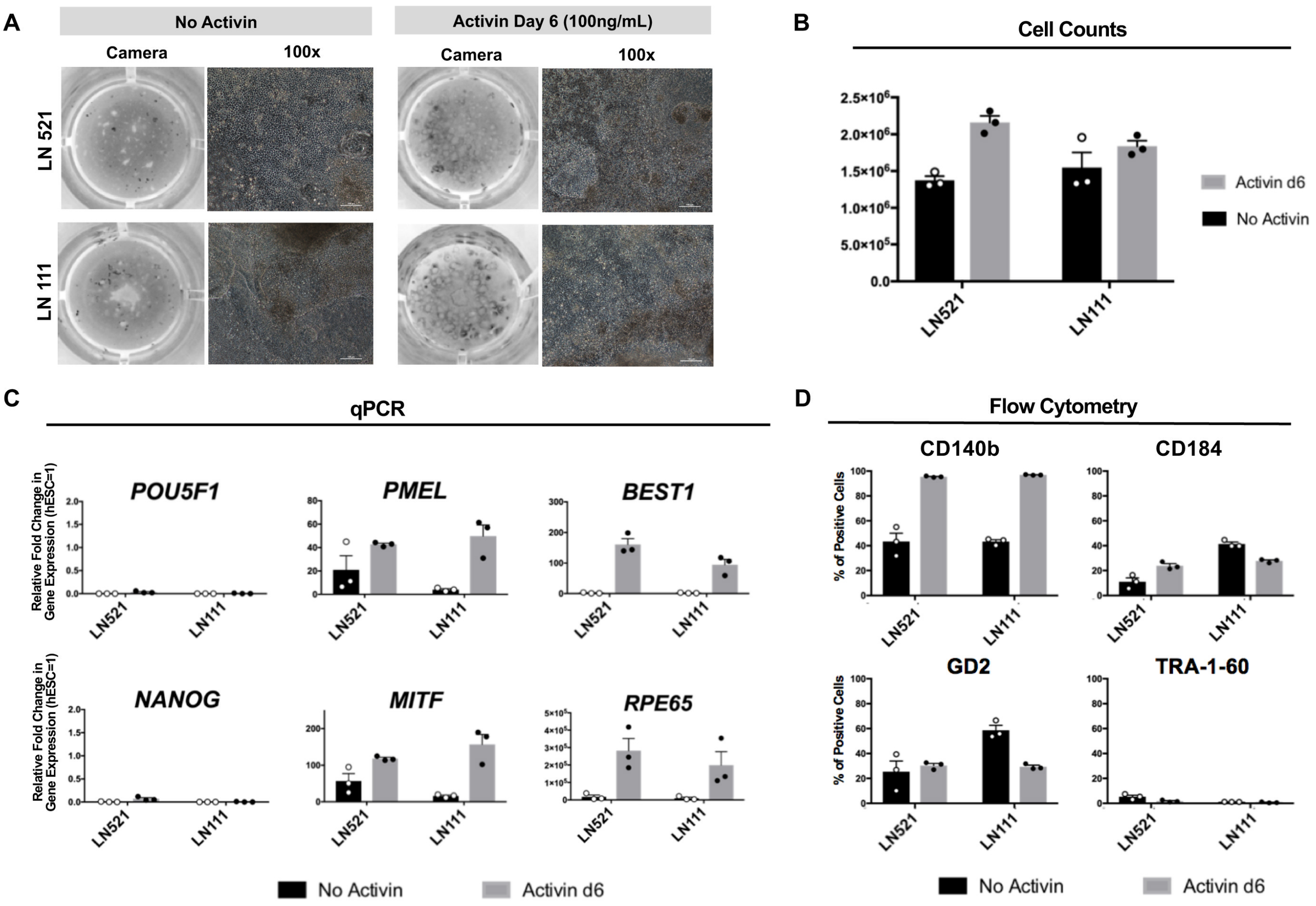

Activin A enhances the efficiency of hPSC-RPE 2D differentiation.

(A) Camera and bright field pictures of hPSC-RPE cultured for 30 days on hrLN-521 or hrLN-111 with or without the addition of Activin A. **(B)** Cell counts at day 30 of differentiation for conditions described in (A). **(C)** Gene expression analysis of pluripotency and RPE-specific genes. Values are normalized to *GAPDH* and displayed as relative to undifferentiated hESC. **(D)** Bar plots showing percentage of positive cells for CD140b, CD184, GD2 and TRA-1-60 measured by flow cytometry on cells cultured on either hrLN-521 or hrLN-111 and with or without the addition of Activin A. Bars represent means ± SEM from three independent experiments. Scale bars: (A) = 100 µm. Source data are provided as a Source Data file.

Supplementary Figure 3

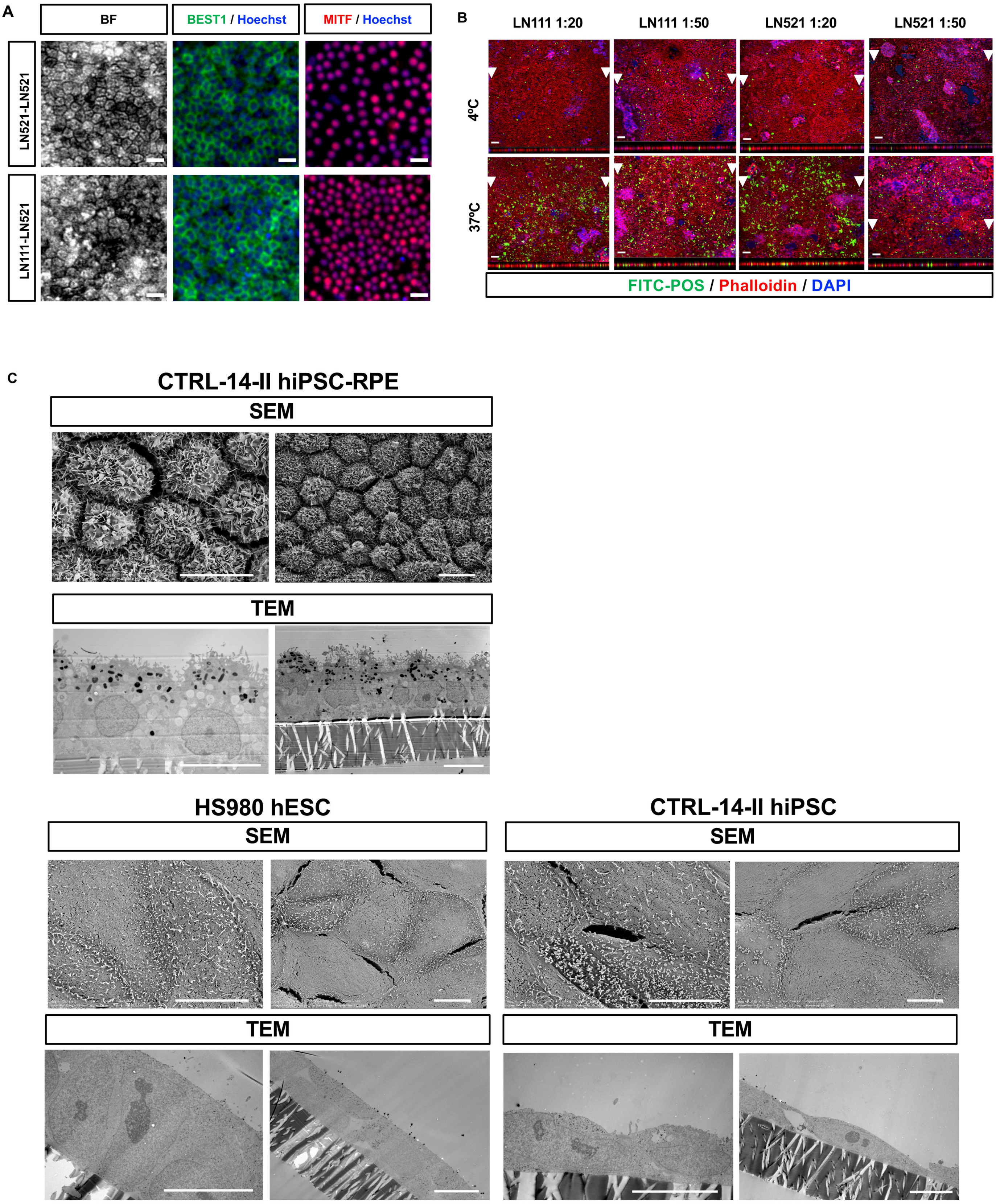

hPSC-RPE are functionally mature after 60 days of differentiation, especially with $7x10^4$ cells/cm^2 (1:20) replating cell density.

(A) Bright field and immunofluorescence pictures showing pigmentation and the expression of RPE specific markers (BEST-1 and MITF) at day 60 on both hrLN-111 and hrLN-521. **(B)** Phagocytosis of FITC-labeled photoreceptor outer segments (POS) by hPSC-RPE induced on hrLN-521 or hrLN-111 and replated at a cell density of $7x10^4$ cells/cm^2 (1:20) or $2.8x10^4$ cells/cm^2 (1:50) on hrLN-521, after overnight incubations at 4°C (negative control) and 37°C. Pictures are a composite of maximum intensity projection (MIP) images depicting internalized FITC-labeled POS in green and phalloidin staining of hPSC-RPE in red, and Z-stack confocal projections of the area delimited by arrows in the MIP. **(C)** SEM and TEM images of hiPSC-RPE, hESC and hiPSC at two different magnifications. Scale bars: (A, B) = 50 µm; (C) 10 µm.

Supplementary Figure 4

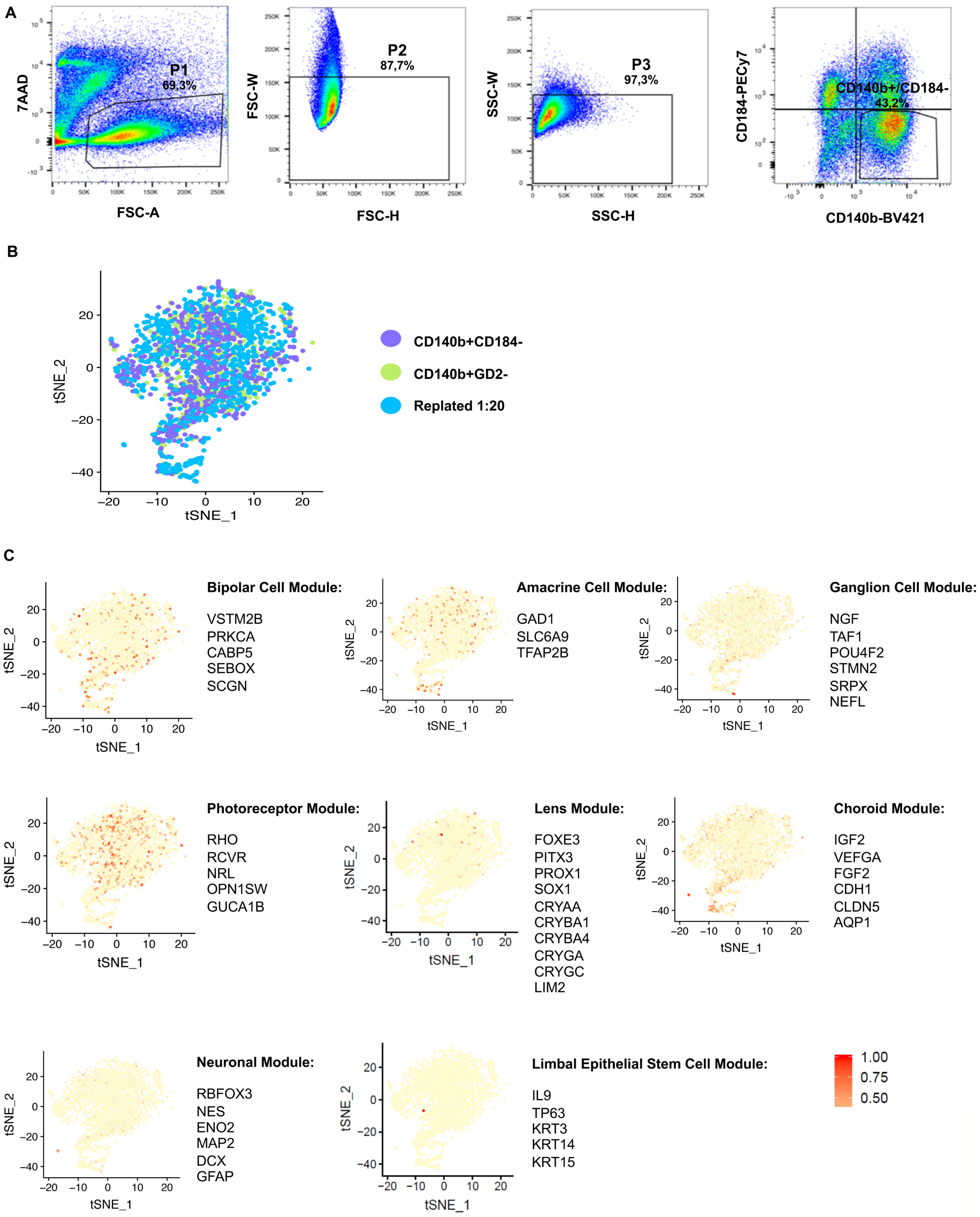

hPSC-RPE cultures show no presence of contaminant cells from other retinal lineages. **(A)** t-SNE plot of all cells showing distribution of the 3 samples analysed: Replated 1:20, CD140b+CD184- and CD140b+GD2-. **(B)** Feature plots displaying expression module scores over the t-SNE plot for distinctive genes for neurons and several retinal cell types: bipolars, amacrine, ganglion cells, photoreceptors, lens, choroid limbal epithelial stem cells. **(C)** Representative gating strategy used for isolation of cell populations profiled in Figure 4.

Supplementary Figure 5

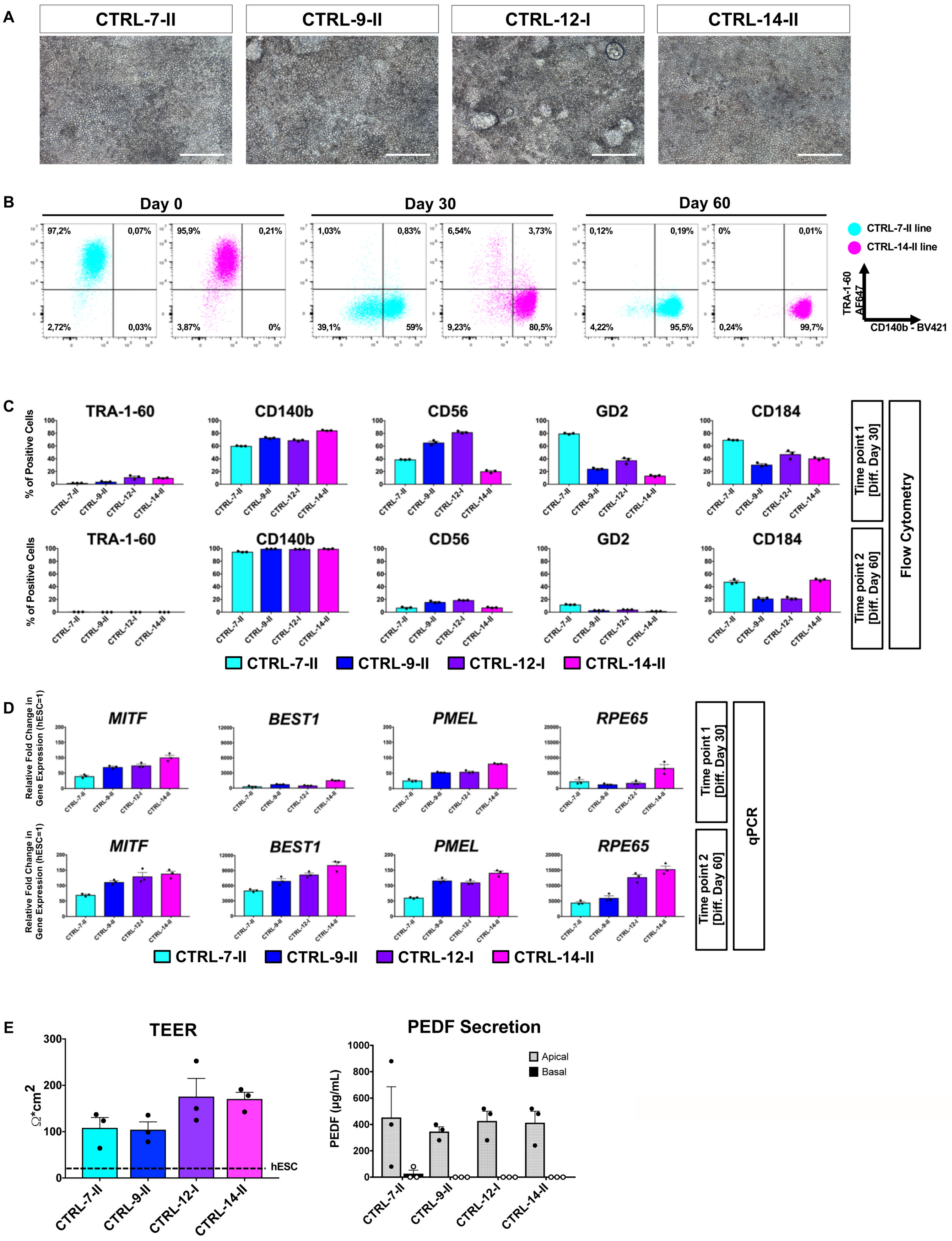

The identified cell surface markers can monitor hPSC-RPE differentiation on hiPSC.

(A) Bright field pictures of CTRL-7-II, CTRL-9-II, CTRL-12-I and CTRL-14-II induced pluripotent stem cell lines after 60 days of differentiation. **(B)** Illustrative dot plots showing TRA-1-60 and CD140b expression intensity measured by flow cytometry at day 0, day 30 and day 60 of differentiation in two of the four induced pluripotent stem cell lines tested (CTRL-7-II and CTRL-14-II)**. (C)** Percentage of positive cells for TRA-1-60, CD140b, CD56, CD184 and GD2 measured by flow cytometry at day 30 and day 60 of differentiation on the four different induced pluripotent stem cell lines. **(D)** Gene expression analysis of RPE genes at day 30 and day 60 of differentiation. Values are normalized to *GAPDH* and displayed as relative to undifferentiated hiPSC. Bars represent means ± SEM from three independent experiments. **(E)** Functional assays demonstrating monolayer integrity measured by transepithelial resistance (TEER), and pigment epithelium-derived factor (PEDF) polarized secretion measured by ELISA after 60 days of hPSC-RPE differentiation. Basal values for all evaluated lines were not detected. The TEER value for undifferentiated hESCs is shown for comparison (dashed line). Bars in all bar graphs represent means±SEM from three independent experiments. Scale bars: (A) = 200 µm. Source data are provided as a Source Data file.

Supplementary Figure 6

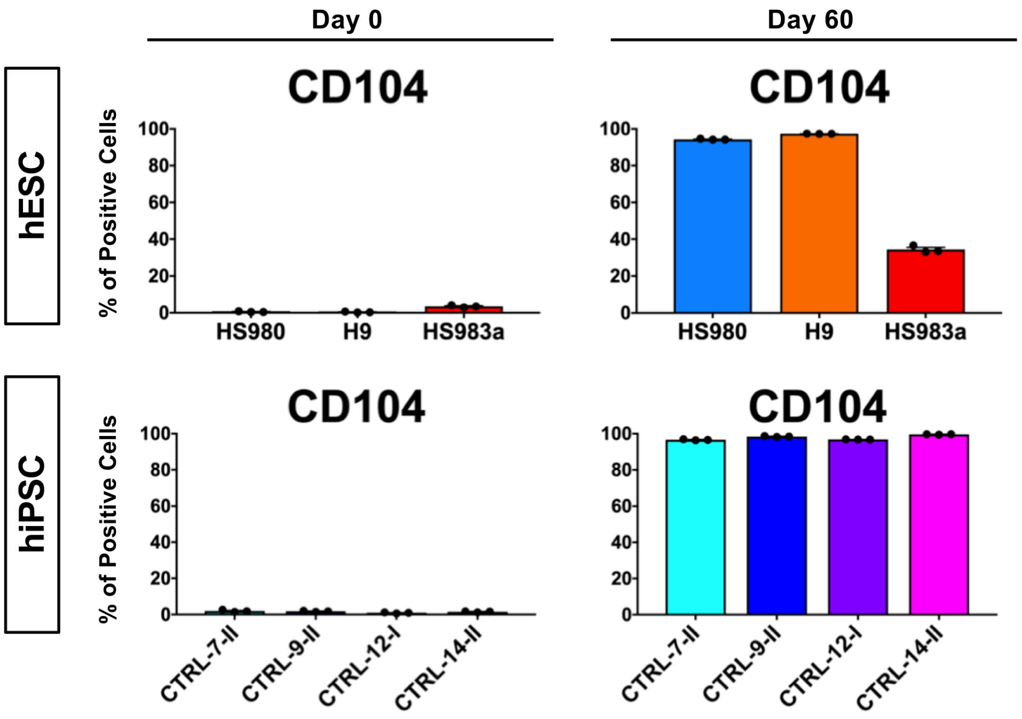

CD104 may be a distinctive cell surface marker of mature hPSC-RPE.

Bar graphs showing the percentage of positive cells for CD104 measured by flow cytometry at day 0 and day 60 of differentiation using three different embryonic stem cell lines and four induced pluripotent stem cell lines. Bars represent means ± SEM from three independent experiments. Source data are provided as a Source Data file.

Supplementary Figure 7

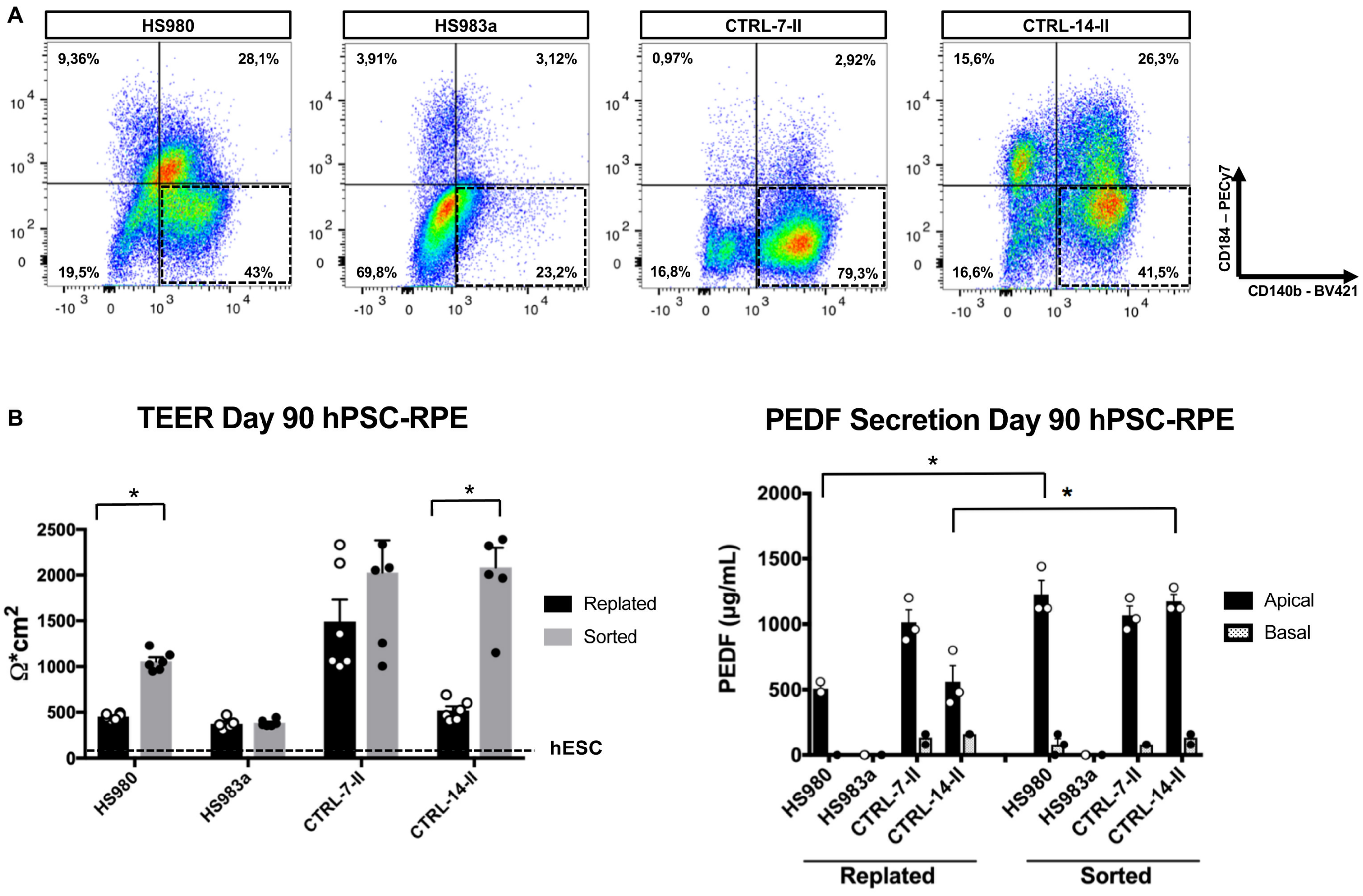

CD140b+ CD184- enrichment improves suboptimal hPSC-RPE differentiations.

(A) Representative flow cytometry dot plot showing gating strategy followed to sort out CD140b+/CD184- hPSC-RPE after 30 days of differentiation in two hESC lines (HS980 and HS983a) and two hiPSC lines (CTRL-7-II and CTRL-14-II). **(B)** Functional assays demonstrating monolayer integrity measured by transepithelial resistance (TEER) and pigment epithelium-derived factor (PEDF) polarized secretion measured by ELISA after 90 days of hPSC-RPE differentiation in the four hPSC-RPE lines tested. Values for HS983a line were below the limit of detection. Bars represent means ± SEM from three independent experiments. (*) Asterisks represent significance with a *P*-value = 0.048 (S7B, TEER, HS980); <0.0001 (S7B, TEER, CTRL-14-II); <0.0001 (S7B, PEDF Secretion, HS980 and CTRL-14-II). Source data are provided as a Source Data file.
