# Supplementary material for: Identification of cell surface markers and establishment of monolayer differentiation to retinal pigment epithelial cells
Source: Nat Commun. 2020 Mar 30;11:1609. doi: 10.1038/s41467-020-15326-5 (PMC7105463; doi:10.1038/s41467-020-15326-5)
Supplement: Supplementary file 4 — Description of Additional Supplementary Files [file 41467_2020_15326_MOESM4_ESM.pdf]

Title: SUPPLEMENTARY DATA 1.
Description: Summary of results for the surface protein screen, related to Figures 1A and 1B.

Title: SUPPLEMENTARY DATA 2.
Description: List and supporting information of the primary and secondary antibodies used in the study, related to Experimental Procedures.
