# Supplementary material for: Identification of cell surface markers and establishment of monolayer differentiation to retinal pigment epithelial cells
Source: Nat Commun. 2020 Mar 30;11:1609. doi: 10.1038/s41467-020-15326-5 (PMC7105463; doi:10.1038/s41467-020-15326-5)
Supplement: Supplementary file 5 — Reporting Summary [file 41467_2020_15326_MOESM5_ESM.pdf]

nature research

Corresponding author(s): Fredrik Lanner

Last updated by author(s): Dec 15, 2019

Reporting Summary

Nature Research wishes to improve the reproducibility of the work that we publish. This form provides structure for consistency and transparency in reporting. For further information on Nature Research policies, see Authors & Referees and the Editorial Policy Checklist .

Statistics

For all statistical analyses, confirm that the following items are present in the figure legend, table legend, main text, or Methods section.

n/a	Confirmed	
☐	☒	The exact sample size (*n*) for each experimental group/condition, given as a discrete number and unit of measurement
☐	☒	A statement on whether measurements were taken from distinct samples or whether the same sample was measured repeatedly
☐	☒	The statistical test(s) used AND whether they are one- or two-sided<br>*Only common tests should be described solely by name; describe more complex techniques in the Methods section.*
☒	☐	A description of all covariates tested
☐	☒	A description of any assumptions or corrections, such as tests of normality and adjustment for multiple comparisons
☐	☒	A full description of the statistical parameters including central tendency (e.g. means) or other basic estimates (e.g. regression coefficient) AND variation (e.g. standard deviation) or associated estimates of uncertainty (e.g. confidence intervals)
☐	☒	For null hypothesis testing, the test statistic (e.g. *F*, *t*, *r*) with confidence intervals, effect sizes, degrees of freedom and *P* value noted<br>*Give P values as exact values whenever suitable.*
☒	☐	For Bayesian analysis, information on the choice of priors and Markov chain Monte Carlo settings
☒	☐	For hierarchical and complex designs, identification of the appropriate level for tests and full reporting of outcomes
☒	☐	Estimates of effect sizes (e.g. Cohen's *d*, Pearson's *r*), indicating how they were calculated

Our web collection on statistics for biologists contains articles on many of the points above.

Software and code

Policy information about availability of computer code

Data collection	10xGenomics: NovaSeq 6000; Smart-seq2: HiSeq 2000; FACS: BD Aria Fusion and FACSDiva software v8.0.1; Flow Cytometry: CytoFLEX S and CytExpert software v2.0.0.153; qPCR machine: StepOneTM real-time PCR System (Applied Biosystems); Image acquisition: Nikon spinning disk confocal microscope with NIS-Elements AR software v5.11.02 and Zeiss LSM710-NLO microscope with ZEN Microscope Software v2.3; ELISA: Softmax Pro v7; SEM: ZEISS GeminiSEM Software; TEM: Hitachi TEM Control Software.
Data analysis	scRNA-seq data: Cell Ranger version 2.1.1 and 3.0.1 for sample demultiplexing, barcode processing, read alignment and single cell 3' gene counting of 10xGenomics seq data; scRNA-seq data analysis was performed using scripts in R (available on request).; flow data analysis: . FlowJo v10 software, Image analysis: Fiji/ImageJ software v2.0 and/or IMARIS v9.5; Phagocytosis assay automated counting: Cell Profiler v3.1; qPCR: Microsoft Excel software v16.34; Graph plotting and statistic analysis: PRISM v7.

For manuscripts utilizing custom algorithms or software that are central to the research but not yet described in published literature, software must be made available to editors/reviewers. We strongly encourage code deposition in a community repository (e.g. GitHub). See the Nature Research guidelines for submitting code & software for further information.

Data

Policy information about availability of data

All manuscripts must include a data availability statement. This statement should provide the following information, where applicable:

- Accession codes, unique identifiers, or web links for publicly available datasets
- A list of figures that have associated raw data
- A description of any restrictions on data availability

All relevant data are available from the authors and the single cell RNA sequencing data is deposited in ArrayExpress (EMBL-EBI) Submission ID/Accession Number: E-MTAB-7742.

Field-specific reporting

Please select the one below that is the best fit for your research. If you are not sure, read the appropriate sections before making your selection.

- [x] Life sciences
- [ ] Behavioural & social sciences
- [ ] Ecological, evolutionary & environmental sciences

For a reference copy of the document with all sections, see nature.com/documents/nr-reporting-summary-flat.pdf

Life sciences study design

All studies must disclose on these points even when the disclosure is negative.

Sample size	Sample size was not determined statistically a priori.<br>10 New Zealand white albino rabbits (provided by Lidköpings rabbit farm, Lidköping, Sweden) aged 5 months, weighing 3.5 to 4.0 kg were used in this study for experiments involving the subretinal transplantation of hPSC-RPE.
Data exclusions	As part of the 10xGenomics scRNA-seq analysis, Cell Ranger quality-control filtered cells (718, 810, 931 and 1129 cell-contaning droplets were captured for CD140b+GD2-, CD140b+CD184-, re-plated 1:20 and hESC samples, respectively) were analyzed in R version 3.5.1 (R Core Team) 38, using Seurat suite version 2.3.439,40. As a further quality-control measure, RPE cells with unique expressed genes (≥ 2000 to ≤ 5000), UMIs (≥ 10000 to ≤ 30000) and percentage of UMIs mapping to MT-genes (≥ 0.025 to ≤ 0.10) were selected. Similarly, hESC cells with unique expressed genes (≥ 2000 to ≤ 8000), UMIs (≥ 10000 to ≤ 80000) and percentage of UMIs mapping to MT-genes (≥ 0.025 to ≤ 0.10). This filtration step resulted in final dataset of 616, 725, 779 and 905 cells for CD140b+GD2-, CD140b+CD184-, re-plated 1:20 and hESC samples, respectively.
Replication	hPSC-RPE differentiation and marker validation was performed in 3 different hESC lines and 4 different hiPSC lines.<br>Three independent experiments were performed for all the experiments involving the use of differentiated hPSC-RPE.<br>The cell surface marker screen was performed once for 3D optic vesicles, and once for hPSC-RPE day 60.<br>For representative images, each experiment was successfully repeated at least three times under similar conditions.<br>All attemps at replication were successful.
Randomization	Animals were randomly assigned to experimental groups. No randomization was performed in our in-vitro experiments.
Blinding	Investigators were not blinded during data collection and analysis.

Reporting for specific materials, systems and methods

We require information from authors about some types of materials, experimental systems and methods used in many studies. Here, indicate whether each material, system or method listed is relevant to your study. If you are not sure if a list item applies to your research, read the appropriate section before selecting a response.

Materials & experimental systems

n/a	Involved in the study	
[ ]	[x]	Antibodies
[ ]	[x]	Eukaryotic cell lines
[x]	[ ]	Palaeontology
[ ]	[x]	Animals and other organisms
[x]	[ ]	Human research participants
[x]	[ ]	Clinical data

Methods

n/a	Involved in the study	
[x]	[ ]	ChIP-seq
[ ]	[x]	Flow cytometry
[x]	[ ]	MRI-based neuroimaging

Antibodies

Antibodies used	Flow Cytometry: Samples were stained with BV421 Mouse Anti-Human CD140b (BD Biosciences 564124, clone [28D4], 10 µg/mL), PE Mouse Anti-Human CD140b (BD Biosciences 558821, clone [28D4], 10 µg/mL), BB515 Mouse Anti-Human CD56 (BD Biosciences 564489, clone [B159], 2.5 µg/mL), Alexa Fluor 647 Mouse Anti-Human TRA-1-60 (BD Biosciences 560850, clone [TRA-1-60], 0.6 µg/mL), BV421 Mouse Anti-Human CD184 (BD Biosciences 562448, clone [12G5], 2.5 µg/mL), BV421 Mouse Anti-Human Disialoganglioside GD2 (BD Biosciences 564223, clone [14.G2a], 2.5 µg/mL), PECy7 Mouse Anti-Human CD184 (BD Biosciences 560669, clone [12G5], 2.5 µg/mL), BV605 Mouse Anti-Human Disialoganglioside GD2 (BD Biosciences 744071, clone [14.G2a], 2.5 µg/mL) and BV605 Rat Anti-Human CD104 (BD Biosciences 744152, clone [439-9B], 2 µg/mL) conjugated antibodies.<br><br>Surface marker screen: BD LyoplateTM Screening Panels

Immunofluorescence: Samples were stained with PAX6 (1:400, Biolegend 901301), NANOG (1:200, ReproCell RCAB003P), Bestrophin 1 (BEST-1) (1:100, Millipore MAB5466), Microphthalmia-Associated Transcription Factor (MITF) (1:200, Abcam ab3201, clone [D5]), Zonula occludens-1 (ZO-1) (1:100, Invitrogen 40-2200), CRALBP (1:250, Abcam ab15051, clone [B2]), PDGFRB (CD140b) (1:100, BD Biosciences, 558820, clone [28D4]), CD56 (1:100, BD Biosciences, 555513, clone [B159]), CXCR4 (CD184) (1:300, Abcam ab1670,) and Ganglioside GD2 (1:200, Santa Cruz Biotechnology sc-53831, clone [14G2a]). Secondary antibodies used: Alexa Fluor 647 donkey anti-rabbit IgG and Alexa Fluor 488 donkey anti-mouse IgG, donkey anti-mouse IgG1 Alexa Fluor 568, donkey anti-mouse IgG2a Alexa Fluor 488 (all of them from Thermo Fisher Scientific, A31573, A21202, A-21124, A-21131 respectively) diluted 1:1000.

Immunohistochemistry: Samples were stained with human nuclear mitotic apparatus protein (NuMA) (1:200, Abcam ab84680), BEST1 (1:200, Millipore MAB5466), CD140b/PDGFRB (1:100, Santa Cruz Biotechnology sc-432) and CD56/NCAM1 (1:100, Santa Cruz Biotechnology sc-7326). Secondary antibodies (Alexa Fluor 555 donkey anti-rabbit IgG A31572 and Alexa Fluor 647 donkey anti- mouse IgG A31571, both from Thermo Fisher Scientific) diluted 1:200.

Validation

All antibodies used are commercially available and have been validated by the manufacturer. For more details on the specific method used for validation of each antibody used in this study, please check supplementary antibody table.

Eukaryotic cell lines

Policy information about cell lines

Cell line source(s)

hESC lines HS980 and HS983 were derived and cultured under xeno-free and defined conditions according to the previously described method [Rodin, S. et al., Nat. Comm. 2014](Swedish Ethical Review Authority: 2011/745:31/3).
The hESC line WA09/H9 was obtained from Wicell.
hiPSC lines CTRL-7-II, CTRL-9-II, CTRL-12-I and CTRL-14-II, kindly provided by Karolinska Institutet iPSC Core facility (Swedish Ethical Review Authority: 2012/208-31/3, 2010/1778-31/4).

Authentication

All of the hESC lines used have been exhaustively tested including karyotpying, gene and protein expression and differentiation before the start of this study.

Mycoplasma contamination

All hESC and hiPSC lines were tested for mycoplasma and all of them were negative.

Commonly misidentified lines
(See ICLAC register)

None of the hESC and hiPSC lines used were listed as commonly misidentified lines in ICLAC register.

Animals and other organisms

Policy information about studies involving animals; ARRIVE guidelines recommended for reporting animal research

Laboratory animals

10 New Zealand white albino rabbits (provided by Lidköpings rabbit farm, Lidköping, Sweden) aged 5 months, weighing 3.5 to 4.0 kg were used in this study. All experiments were conducted in accordance with the Statement for the Use of Animals in Ophthalmic and Vision Research.

Wild animals

The study did not involve wild animals

Field-collected samples

The study did not involve samples collected from the field

Ethics oversight

The Northern Stockholm Animal Experimental Ethics Committee approved the use of rabbits for studies involving subretinal transplantation of hPSC-RPE.

Note that full information on the approval of the study protocol must also be provided in the manuscript.

Flow Cytometry

Plots

Confirm that:

- [x] The axis labels state the marker and fluorochrome used (e.g. CD4-FITC).
- [x] The axis scales are clearly visible. Include numbers along axes only for bottom left plot of group (a 'group' is an analysis of identical markers).
- [x] All plots are contour plots with outliers or pseudocolor plots.
- [x] A numerical value for number of cells or percentage (with statistics) is provided.

Methodology

Sample preparation

hPSC-RPE and hPSC growing on the tested substrates were dissociated into single cells using TrypLE Select. Cells were incubated with the conjugated antibodies on ice for 30 min. Prior to analysis cells were incubated with a live/dead stain (7AAD or DRAQ7).

Instrument	Stained cells were analyzed using a CytoFLEX flow cytometer equipped with 488 nm, 561 nm, 405 nm and 640 nm lasers (Beckman Coulter) or a LSRFortessa equipped with 405 nm, 640 nm, 488 nm, 355 nm and 561 nm lasers (BD Biosciences).<br>Cell sorting experiments were performed using a BD FACS Aria Fusion Cell Sorter (BD Bioscience).
Software	Analysis of the data was carried out using FlowJo v.10 software (Tree Star).
Cell population abundance	When cells were sorted based on CD140b signal at day 30 of differentiation, a CD140b+ cell population of 85 – 95% was observed.<br>When cells were sorted based on CD140b and CD184 signal at day 30 of differentiation, a CD140b+ / CD184- cell population of 40 – 80% was observed.<br>When cells were sorted based on CD140b and GD2 signal at day 30 of differentiation, a CD140b+ / GD2- cell population of 70 – 80% was observed.
Gating strategy	Cells were gated based on FSC-A/SSC-A for P1 and FSC-W/FSC-H (P2) and for singlets. Dead cells were then excluded from the analysis by gating for 7AAD or DRAQ7 negative population (P3).<br>Fluorescence minus one (FMO) controls or undifferentiated hPSC were included for each condition to identify and gate negative and positive cells. Representative plots for the gating strategies used in each experiment were included in Supplementary Figure 8.

☒ Tick this box to confirm that a figure exemplifying the gating strategy is provided in the Supplementary Information.
